# Drift, selection, or migration? Processes affecting genetic differentiation and variation along a latitudinal gradient in an amphibian

**DOI:** 10.1186/s12862-017-1022-z

**Published:** 2017-08-14

**Authors:** Maria Cortázar-Chinarro, Ella Z. Lattenkamp, Yvonne Meyer-Lucht, Emilien Luquet, Anssi Laurila, Jacob Höglund

**Affiliations:** 1 Animal Ecology/Department of Ecology and Genetics, Uppsala University, Norbyvägen 18D, 75236 Uppsala, Sweden; 2 Present address: Department of Neurogenetics of Vocal Communication, Max Planck Institute of Psycholinguistics, Box 310, 6500 Nijmegen, Netherlands; 3 Present address: Université Claude Bernard - Lyon I, CNRS, UMR 5023 – LEHNA, 3–6, rue Raphaël Dubois - Bâtiments Darwin C and Forel, 69622 Villeurbanne Cedex 43, Boulevard du 11 novembre, 1918 Lyon, France

**Keywords:** Genetic drift, Natural selection, Major histocompatibility complex, Microsatellites, Outlier tests, *Rana arvalis*

## Abstract

**Background:**

Past events like fluctuations in population size and post-glacial colonization processes may influence the relative importance of genetic drift, migration and selection when determining the present day patterns of genetic variation. We disentangle how drift, selection and migration shape neutral and adaptive genetic variation in 12 moor frog populations along a 1700 km latitudinal gradient. We studied genetic differentiation and variation at a MHC exon II locus and a set of 18 microsatellites.

**Results:**

Using outlier analyses, we identified the MHC II exon 2 (corresponding to the β-2 domain) locus and one microsatellite locus (RCO8640) to be subject to diversifying selection, while five microsatellite loci showed signals of stabilizing selection among populations. STRUCTURE and DAPC analyses on the neutral microsatellites assigned populations to a northern and a southern cluster, reflecting two different post-glacial colonization routes found in previous studies. Genetic variation overall was lower in the northern cluster. The signature of selection on MHC exon II was weaker in the northern cluster, possibly as a consequence of smaller and more fragmented populations.

**Conclusion:**

Our results show that historical demographic processes combined with selection and drift have led to a complex pattern of differentiation along the gradient where some loci are more divergent among populations than predicted from drift expectations due to diversifying selection, while other loci are more uniform among populations due to stabilizing selection. Importantly, both overall and MHC genetic variation are lower at northern latitudes. Due to lower evolutionary potential, the low genetic variation in northern populations may increase the risk of extinction when confronted with emerging pathogens and climate change.

**Electronic supplementary material:**

The online version of this article (doi:10.1186/s12862-017-1022-z) contains supplementary material, which is available to authorized users.

## Background

The relative roles of selection and drift shaping genetic diversity among and within natural populations are a contentious issue in evolutionary biology [1, 2]. Generally, the force of genetic drift is inversely related to population size, while the opposite is true for selection [3]. At the molecular level, the most common form of selection is purifying selection (either favouring or disfavouring allelic variants) which leads to loss of genetic variation. However, genetic loci can also be under various forms of balancing selection, which uphold and maintain variation over space and time [4]. As the effect of genetic drift is imminently linked with population size, variation in population size within a species’ range is expected to play a central role for the importance of genetic drift. As species are generally expected to achieve their highest abundances at the center of their range, and populations tend to become progressively smaller towards the edge of the range (the ‘abundant centre’ model; [5, 6]), drift is considered as more important in populations closer to the range edge, leading into reduced genetic variation and stronger population structure in these peripheral populations [7, 8].

While the abundant center model has been influential for much of the theory, evidence for populations being more numerous or frequent at the centre of the distribution is, at best, limited [5]. Here, historical events like colonization processes can have a significant influence on patterns of genetic variation [8]. When new populations are repeatedly founded by a few individuals these founder effects will lead to loss of genetic variation [9–11]. In Northern Europe, the climate was considerably colder in the northern hemisphere during the Pleistocene, and most of the species currently present had withdrawn to refugia in Southern Europe [12, 13]. After the retreat of the glaciers, species expanded and recolonized the formerly uninhabitable areas [14, 15]. During the Holocene recolonization genetic variation was lost due to repeated founder events. As a result, less diverse populations are often found at northern latitudes [16–18], and these populations are predicted to genetically differ from the southern ones due to their demographic history.

Disentangling whether current patterns of genetic variation along geographical gradients are brought about by central-marginal or colonization processes is not simple, because, due to the location of glacial refugia, the two patterns are geographically correlated [8]. Moreover, these processes can be complicated by historical processes when populations following different migration routes meet in a secondary contact zone [8] and confounded by the fact that divergent selection in different parts of the species range may also cause population divergence [14]. However, such information is pivotal for understanding the genetic processes in contemporary populations.

In population and conservation genetics, spatial population structure and gene flow are usually assessed with neutral markers, assuming that this variation also reflects adaptive potential [19] However, the use of only neutral markers has important limitations and does not provide a complete picture on genetic variation and the evolutionary potential of wildlife populations [20–23]. In contrast to neutral markers, loci of the Major Histocompatability Complex (MHC) comprise well understood coding genes which show a high level of variation [24–28]. The MHC is a multigene family and the most polymorphic functional set of loci in vertebrate genomes described so far [29–33]. It codes for proteins that are part of the adaptive immune system and associated with disease and parasite resistance [4, 34–36]. Previous studies have shown a direct association between specific MHC alleles and pathogen resistance (e.g. [24, 37–40]). However, other studies have claimed a lack of association between disease resistance and MHC genetic variation [41, 42].

Amphibians are globally subjected to severe declines and currently the most threatened vertebrate class [43]. Along with the severe impact of habitat fragmentation, emerging infectious diseases are accounting for the global decline such as the chytridiomycosis caused by the chytrid fungus *Batrachochytrium dendrobatidis* (*Bd*), which has caused a large number of local and global amphibian extinctions [44, 45]. Several studies have emphasized the importance of MHC variation for disease resistance in amphibians [46]. For example, transcriptomic studies show that *Bd* infection activates immune pathways in many species [47, 48], and MHC heterozygous individuals or individuals with unique MHC alleles have been shown to be resistant to *Bd* [38, 39, 49] and other pathogens [50, 51]. The study of MHC variation is especially important in amphibian populations at northern latitudes, where genetic variation is often lower due to postglacial colonization processes. Indeed earlier studies in two amphibian species (*Triturus cristatus*: [52]; *Epidalea calamita*: [53, 54]) have found low MHC variation in high-latitude populations, potentially rendering these populations especially vulnerable to disease. With these exceptions, little is known about how adaptive genetic variation in general, and MHC variation in particular, varies with latitude in amphibians.

Studies on life-history variation of ranid frogs in northern Europe have revealed extensive adaptive genetic divergence in larval development rates, both at local scale (*Rana temporaria*; *Rana arvalis;* [55–57] and over wider latitudinal gradients (*Rana temporaria; Pelophylax lessonae* [58, 59]. Here the thyroid hormone pathway has an important dual function repressing thyroid-induced gene expression in premetamorphic tadpoles and, on the other hand, activating thyroid-induced genes to initiate metamorphosis (*Xenopus laevis;* [60, 61]). The microsatellite locus RCO8640 located inside the up-regulated transcription factor gene C/EBP is involved in the thyroid hormone pathway (*Lithobates catesbiana*; [62, 63], and has been linked with adaptive divergence in larval developmental rate (*Rana arvalis*; [56, 57]) providing an additional marker of adaptive genetic variation.

In this study, we assessed genetic variation in 12 moorfrog *Rana arvalis* populations along a 1700 km latitudinal gradient from northern Germany to northern Sweden. Sampling along this gradient allowed us to study variation in the single MHC class II exon 2 locus and 18 microsatellites in order to: 1) trace the postglacial history of the moor frog along the gradient, 2) study the level of population genetic diversity along the gradient in order to elucidate adaptability to more local environments, and 3) investigate possible different effects of selection along the gradient. Our ultimate goal is to understand how these processes combine to create a complex pattern of within and among site genetic diversity of *R. arvalis* along the latitudinal gradient.

## Results

### Microsatellites – Null alleles and detection of loci under selection

**Fig. 1:**
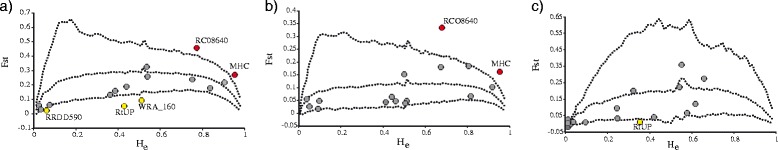
F_ST_ vs expected heterozygosity for each 15 microsatellite and the MHC II exon 2 locus. Black dashed lines show the upper and lower 99% confidence intervals with 10,000 simulations from a stepwise mutation model (SMM), loci under neutrality expectations are colored in grey, loci under differential selection are colored in yellow and loci under diversifying selection are colored in red. Figure **a** represent the plot for the entire gradient, figure **b** the southern cluster and figure **c** northern clusters, respectively

Out of 28 initially tested microsatellite loci, three (Rtempμ9, Rtempμ7, Rt2Ca9) were monomorphic, three (WRA1–22, WRA1–28, WRA6–8) failed to amplify and four (RtμJ, RNTYR2, RNTYR2, RtCa11) produced ambiguous scoring patterns and were therefore excluded from subsequent analyses (Additional file 1: Table S1). The remaining 18 microsatellites were polymorphic in all populations with 6.3 alleles per locus on average. Three out of the remaining 18 loci (EU_11, RECALQ and RtCa41) were found to contain null alleles and were removed from further analyses. Five loci (RRDD590, Rtca18, RtuP, RtCa25, and WRA_160) were identified to be under stabilizing selection with F_ST_ lower than 0.03, nine loci were identified as neutral (R1atCa17, Rtempμ4, Rtempμ5, RCIDII, EU_06, EU_12, EU_15, EU_19, EU_24), whereas the locus RCO8640 was under diversifying selection with high F_ST_ (0.48; Additional file 1: Table S1 and Additional file 2: Table S2). All loci identified as outliers by Lositan and/or BayeScan were deemed as “under selection” and excluded from the neutral expectation analyses (Additional file 2: Table S2). All loci that were under diversifying selection, stabilizing selection, identified either by Lositan or Bayescan, or neutral expectations when the MHC II exon 2 was included in the analyses are summarized in Additional file 3: Table S3. The MHC II exon 2 and RCO8640 were under diversifying selection in the full gradient and in the southern cluster but not in the northern cluster (Fig. 1, Additional file 3: Table S3).

### MHC II exon 2 diversity: Miseq run summary

We obtained a total of 20 million of MiSeq sequence reads in two separate runs, an average of 2.5–2.7 million reads per pool with intact primers and attached barcode information that could be assigned to a total of 361 amplicons from 207 individuals. In total 33 samples out of 240 failed due to PCR amplification problems. The average number of reads per amplicon was 13,404, ranging from 300 to 53,006; five amplicons with <300 reads were discarded. We amplified and sequenced 111 of the 207 genotyped individuals in two or three replicates, which corresponds to a replication rate of 53.6%, including every amplicon that revealed a unique MHC allele. Replicates were randomly assigned across different pools to avoid false allele identification. All replicates revealed the identical genotype as the respective original sample, leading to a genotyping reproducibility of 100%. Among the 207 individuals, we assigned 57 valid MHC II exon 2 alleles with a length of 272 bp and 27 polymorphic nucleotide positions. None of these sequences showed insertions, deletions or stop codons, therefore we assume that these are true alleles. All the 57 valid MHC II exon 2 allele sequences were translated into unique amino acid alleles. We checked for signs of recombination by using Omegamap [64] and did not find any signal of recombination in MHC class II exon 2. By following the DOC method [65], we detected a single MHC class II locus in 206 individuals. One sample (A10; Germany), however, revealed a second MHC class II locus in apparently lower read numbers in two of the three replicates, pointing to the possible existence of a very rare MHC class II duplication. We conclude that we are working with a single MHC class II locus in our data set.

### Genetic structure among populations

The neutral microsatellites markers showed a global *F*_ST_ of 0.19 (95% C.I. = 0.14–0.26). In the northern cluster (see below) *F*_*ST*_ for neutral microsatellites was 0.12 (95% C.I. = 0.05–0.16) and in the southern cluster 0.09 (95% C.I. = 0.00–0.15). Population differentiation increased with geographic distance, showing clear and significant IBD (R^2^ = 0.54; *p* = 0.003; Additional file 4: Figure S1). Using the STRUCTURE admixture model, the most likely number of genetic clusters was two (K = 2), revealing a northern (Umeå and Luleå) and a southern cluster (Uppsala, Skåne and Germany). Further STRUCTURE runs suggested population substructure (Additional file 5: Figure S2). The Uppsala and Germany populations seemed to be more similar to each other than to the Skåne populations when K = 3 was modelled. Four different clusters were observed when K = 4 was modelled while the same four clusters were observed with K = 5 since the northern populations (Luleå and Umeå) were not differentiated. A similar result was also found in the DAPC analyses which revealed two well defined clusters (a southern and a northern) with further substructure in concordance with the STRUCTURE results (Additional file 6: Figure S3). We did not find any evidence of gene flow between any of populations in the entire gradient using divMigrate [66]. This was the case even among local populations within the regions.

**Fig. 2:**
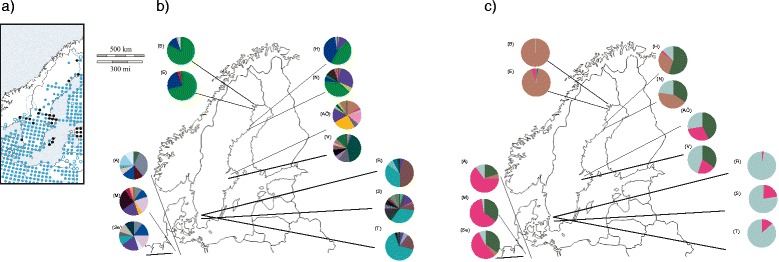
**a** Approximate distribution of *R. arvalis* in Europe. The map is based on information in Gasc et al. [116] (*blue dots* and *open circles*), and updated with information from the Swedish Species Information Centre (http://artfaktaartdatabankense/taxon/208250), Sillero et al. [84], and own observations (*black dots*). **b** Allelic distribution of MHC Class II alleles and **c** RCO8640 in 12 *R arvalis* populations (B: Besbyn (Luleå); F: Ernäs (Luleå); N: Nydalasjön (Umeå); H: Holmsjön (Umeå); ÖA: Österbybruk (Uppsala); V: Valsbrunna (Uppsala); R: Räften (Skåne); S: Sjöhusen (Skåne); T: Tvedöra (Skåne), M: Mardorf (Germany), Se: Seebeckwiesen (Germany). A: Altwarmbüchen (Germany)). Colour coding scheme for MHC alleles is given in the (Additional file 5: Fig. S2)

The global *F*_ST_ for MHC II exon 2 in the entire gradient was 0.36 with the German populations included (95% C.I. = 0.32–0.40) and 0.42 (95% C.I. = 0.23–0.47) when excluded. We found significant IBD (R^2^ = 0.53; *p* = 0.031; Additional file 4: Figure S1). F_ST_ was 0.14 (95% C.I. = 0.07–0.18) and 0.28 with the German populations included (95% C.I. = 0.21–0.33) within the northern and southern cluster, respectively and 0.35 in the south when German population were excluded (95% C.I. = 0.24–0.42). The populations were clearly differentiated and genetically structured (Additional file 7: Table S4). Allele frequency pie-charts showed a structured pattern from northern to southern populations forming two clearly different clusters - a northern (Luleå and Umeå) and a southern (Uppsala, Skåne and Germany) cluster - for the MHC gene (Fig. 2; Additional file 8: Figure S4). *F*_*ST*_ between these two clusters was 0.42 (*p* < 0.001). Surprisingly, we found several alleles at very low frequencies at intermediate latitudes (Uppsala) and only five alleles were shared between Umeå in the north and the southern populations (Fig. 2; Additional file 8: Figure S4 and Additional file 9: Table S5).

The outlier microsatellite locus RCO8640 showed strong differentiation along the gradient with an overall *F*_*ST*_ of 0.45 (95% C.I. = 0.35–0.55). F_ST_ was 0.33 (95% C.I. = 0.20–0.47) and 0.32 (95% C.I. = 0.23–0.45) within the northern and southern cluster, respectively. Four different alleles were found at this locus. In Luleå, the northernmost populations in the gradient, only one allele was present, and in Umeå this was the most common allele (Fig. 2; Additional file 8: Figure S4).

**Fig. 3:**
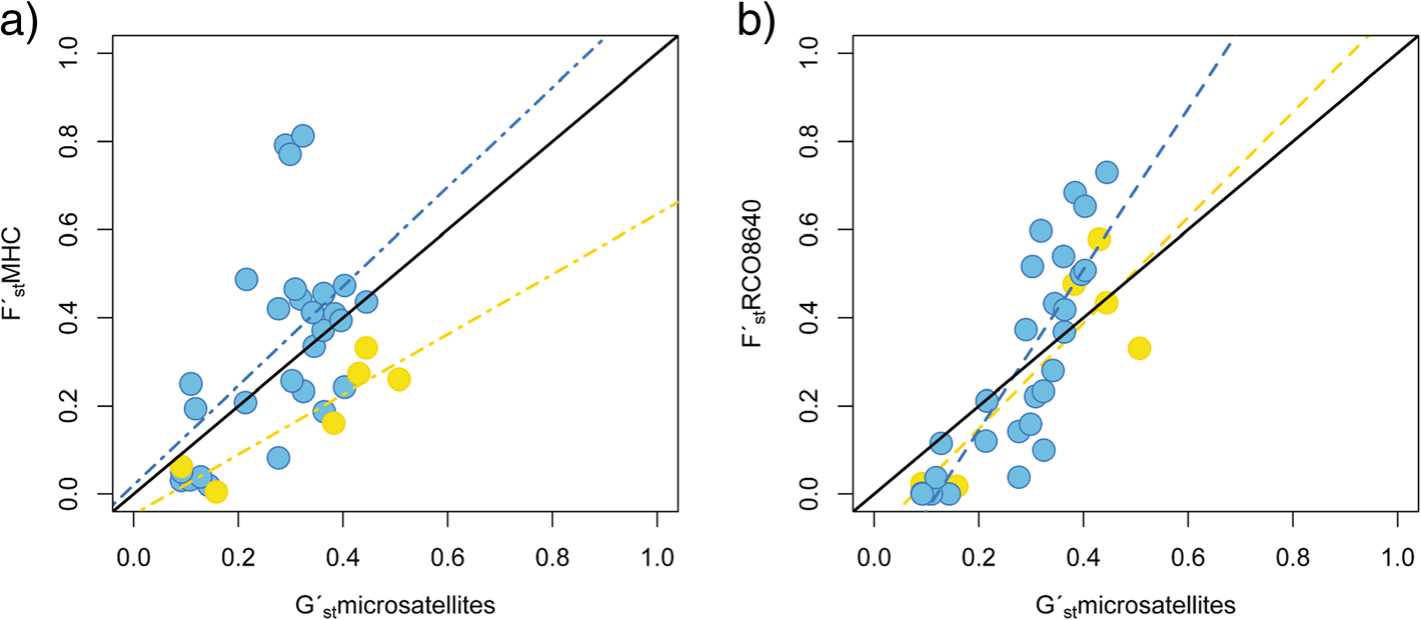
Standardized F’_ST_ pairwise comparisons **a**) for MHC class II and **b**) RCO860 microsatellite marker **b**) in relation to Standardized G’_ST_ pairwise values for neutral microsatellites. The northern cluster is represented by *yellow circles* and the southern cluster is represented by *blue circles*

When comparing differentiation in loci under diversifying selection (MHC and RCO8640) and neutral microsatellites we found that the Restricted Major Axis (RMA) regression slope for MHC differentiation (F’_ST_) against neutral differentiation (G’_ST_) tended to be higher in the southern (slope = 1.89 (95% C.I.: 0.93–3.55) compared to the northern cluster (slope = 0.76 (95% C.I.: 0.35–1.59, Fig. 3) possibly indicating stronger differentiation on MHC in the south. The corresponding RMA slopes for RCO8640 were 2.19 (C.I.: 1.68–2.85) in the southern and 1.41 (C.I.: 0.52–4.00) in the northern cluster (Fig. 3), again suggesting a trend of stronger differentiation in the south although the confidence intervals of the RMA slopes were widely overlapping.

### Genetic diversity within populations

**Fig. 4:**
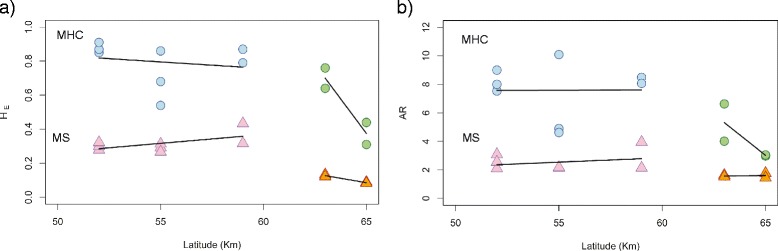
MHC genetic variation (*blue circles* (southern cluster). and *green circles* (northern cluster). Microsatellite variation is given in *pink tringles* (southern cluster) and *orange triangles* (northern cluster). The linear regression is represented by a *black line*. **a**) H_E_ = Expected heterozygosity and **b**) AR = allelic richness

For the nine neutral microsatellites, H_E_ within populations ranged from 0.08 to 0.32 (overall H_E_ = 0.28) and allelic richness from 1.45 to 3.0 (overall AR = 2.22). Observed and expected heterozygosity were not significantly different, as would be expected of selectively neutral loci. We found higher H_E_ and AR values in the southern (i.e., Uppsala and southwards) than in the northern regions (Umeå and Luleå; Table 2). We did not find any significant relationships between latitude and H_E_ or latitude and AR, along either part of the gradient (Fig. 4). The maximum number of alleles was found in the Uppsala region (46) followed by Germany (45).

Across all 207 individuals we found 57 alleles at the MHC class II β locus (Fig. 2; Additional file 8: Figure S4). The number of alleles per population varied substantially among regions. Levels of expected heterozygosity within populations for the MHC locus ranged from 0.31 to 0.91 (overall H_E_ = 0.71) and allelic richness ranged from 2.98 to 10.09 (mean AR = 6.45; Table 1). Levels of observed heterozygosity within populations ranged from 0.30 to 0.90 (overall H_O_ = 0.56; Table 1). Observed and expected heterozygosity were significantly deviant in five out of 12 populations (HWE equilibrium tests; *p* < 0.001). Especially, the German populations (A, M and Se) showed lower H_O_ values than expected (H_O_ = 0.35, H_E_ = 0.85; H_O_ = 0.30, H_E_ = 0.87 and H_O_ = 0.50, H_E_ = 0.91, respectively) compared to the rest of populations (Table 1). These low values of observed heterozygosity might be the result of an amplification problem of some alleles in these populations, leading to an artificial increase in homozygotes. The northern regions (Umeå and Luleå) showed lower diversity than the southern (all remaining) populations in terms of H_E_ and AR (Fig. 4; Wilcoxon test AR; *p* < 0.001 HE; *p* < 0.001). There was no significant relationship between latitude and H_E_ and AR in the southern part of the gradient. However, along the northern part of the gradient H_E_ was lower in the northernmost region (Luleå, Fig. 4 and Table 1). The highest number of alleles (46) was again found within the Uppsala region. Within the entire gradient, 37 out of 57 alleles were private to a single population (Table 1). The number of private alleles within a sampling area was highest in A (Germany, PA = 12) and ÖA (Uppsala, PA = 15) populations (Additional file 9: Table S5). Only one allele (Raar_DAB*15) was widespread and observed in 9 populations from different regions, while two alleles (Raar_DAB*20 and Raar_DAB*21) were only shared between the 4 populations in northern Sweden (Umeå and Luleå regions; Fig. 2; Additional file 8: Figure S4; Additional file 9: Table S5).

**Table 1:** Genetic variation at the MHC II exon 2 locus in the populations

Locality	Sampling area	code	n	NA	As	PA	H_o_	H_e_	AR
Altwarmbüchen		A	14	8		3	0.35^*^	0.85	7.53
Mardorf	Germany	M	10	8	17	3	0.30^*^	0.87	8.00
Seebeckwiesen		Se	10	9		2	0.50^*^	0.91	9.00
Sjohusen		R	20	14		2	0.80	0.68	4.90
Tvödöra	Skåne	S	20	6	18	7	0.90	0.86	10.09
Räften		T	19	6		1	0.42^*^	0.54	4.62
Österbybruk		AÖ	18	11		5	0.83	0.87	8.49
Valsbrunna	Uppsala	V	19	11	19	7	0.78	0.79	8.07
Holmsjön		H	19	5		0	0.57	0.64	4.01
Nydalasjön	Umeå	Ny	19	9	10	3	0.57^*^	0.76	6.63
Besbyn		B	20	4		2	0.30	0.31	2.98
Ernäs	Luleå	E	19	4	6	2	0.42	0.44	3.05
Total			207			37	0.56	0.71	6.45

The populations are ordered from South to North. n = number of individuals; NA = alleles within a population; As = alleles within a sampling area; PA = private alleles; H_o_ = observed heterozygosity, H_e_ = expected heterozygosity; AR = allelic richness. The HO that deviate significantly from H-W expectations are marked with a ^*^

## Discussion

We studied the MHC and microsatellite genetic variation in *R. arvalis* populations along a 1700 km latitudinal gradient and assessed the relative contributions of drift, selection and migration/colonization to understand the postglacial colonization history and the evolutionary forces acting on the adaptive potential and genetic variation of the populations. Four main results can be derived from our analyses. First, the postglacial migration history has resulted in two major clusters currently present in northern Germany and the Scandinavian Peninsula: a northern and a southern. Second, within population genetic variation is higher in the southern as compared to the northern cluster for all the studied genetic markers. Third, there are indications that selection is likely weaker and drift stronger in the northern cluster. Fourth, these forces combined have led to a complex pattern of differentiation along the gradient where some loci are more divergent among populations than predicted from drift expectations due to diversifying selection, while other loci are more uniform among populations due to stabilizing selection. We will discuss each of these conclusions in detail below.

### Postglacial colonization history

Previous phylogeographical studies of *R. arvalis* suggest two routes of postglacial recolonization from south-eastern Europe to Scandinavia [67, 68]. Accordingly, *R. arvalis* expanded from refugia in the Balkans and southern Russia, and used two different postglacial recolonization routes to colonize the Scandinavian Peninsula; one crossing from western central Europe to Sweden from the south-west via the postglacial land bridge that connected Denmark and Sweden, and the other using a route on the eastern side of the Baltic Sea via Finland to northern Sweden [67, 68]. Under this scenario the two lineages met in northern Sweden forming a contact zone, which exact location remains to be formally identified. Our results are in accordance with the previous studies. However, our study suggests that the contact zone lies further south (i.e. between Uppsala and Umeå) than the previous, primarily mt-DNA based, analysis [68], which placed the contact zone between Umeå and Luleå.

### Within region diversity

Our results show lower genetic variation for both neutral microsatellites and the MHC II exon 2 locus in populations from the north of Sweden as compared to southern populations. For the MHC II exon 2 locus this difference is especially pronounced in the northernmost sampling region (Luleå), where we only found six of the 57 alleles present along the gradient. These results are in concordance with studies on different taxa, which have found lower genetic variability in northern Europe as compared to central European populations [13, 16], and this is usually interpreted as a consequence of northern Sweden being the last area in Europe to be recolonized after the last glaciation events. This is also what is predicted by the central marginal hypothesis of genetic variation [7, 8]. However, in the case of *R. arvalis*, our analyses showed that the observed clinal variation is not simply due to a gradual decline along the colonization gradient as the two lineages colonizing the Scandinavian Peninsula meet at intermediate latitudes somewhere between Uppsala and Umeå. This leads to a prediction that these two localities are the least diverse along each of the respective colonization routes. However, the northernmost locality, Luleå, is the one showing the lowest within site diversity.

The depletion in genetic variation observed in the north suggests lower adaptive potential in response to climate change in northern populations [69]. The reduced variation in MHC exon II in the northern cluster is in line with the earlier results on two amphibian species [52–54]. Although these results suggest long-term survival of populations with very low MHC variation in postglacial expansion areas for hundreds of generations, the low number of MHC II alleles in the northern population (Luleå) can be a disadvantage in the future if the populations are confronted with novel or emerging diseases [70, 71].

The high levels of expected heterozygosity, the large number of rare alleles - numbers exceeding or being in line with what has previously been reported for refugial populations in unglaciated areas of Europe [53] – as well as the heterozygosity excess for the MHC exon II in some southern populations might be explained by two hypotheses on pathogen mediate-selection mechanisms (PMS). First, *the heterozygote advantage hypothesis* assumes that heterozygous individuals are favored because they can recognize a broader range of pathogens [72, 73]. However, published evidence confirming a MHC-specific heterozygote advantage is limited [38, 74, 75]. Second, the *rare allele advantage hypothesis* assumes that uncommon alleles in the populations are likely to offer more protection than common alleles and thus confer a selective advantage [76]. In our data, we found rare alleles in almost all the populations over the entire gradient. These rare alleles could be a potential source for defense against pathogens in these populations. With our data we cannot distinguish among these two hypotheses and they are not mutually exclusive [35, 38, 39, 77, 78]. Further investigation regarding allele frequency distributions and parasite infection are needed to understand which mechanisms are responsible for maintaining genetic variation in relation to parasite resistance.

### Selection/drift patterns along the gradient

*R. arvalis* populations showed clear structure and IBD in both adaptive and neutral markers despite being recently diverged in evolutionary time. Outlier analyses suggested that the MHC II exon 2 and RCO8640 loci have been affected by different evolutionary processes in the northern and southern cluster. We saw signs of diversifying selection only in the southern populations while all markers seem to be mainly influenced by drift processes in northern populations. However, this pattern could be enforced by the fact that we studied fewer populations in the northern cluster. A study in Scandinavian common frog *R. temporaria* found that the impact of drift was higher in northern than in the southern populations probably because the northern populations were smaller and more isolated [79]. In our study, *R. arvalis* is at the northernmost range of its distribution and we suggest that populations are smaller and the connectivity among populations is poor in the northern cluster. Therefore, drift processes are likely to be more important at high latitudes due to a high degree of population fragmentation and low effective population sizes (even though we cannot find a clear difference concerning effective sizes among southern and northern populations, Table 2).

**Table 2:** Genetic variation at 9 neutral microsatellites in the studied populations

Locality	Sampling area	code	n	NA	As	PA	H_o_	H_e_	AR	Ne (LDNe)	Ne (CNe)
Altwarmbüchen		A	20	38		8	0.38	0.28	3.08	1.9 (1.0–1.9)	0.7 (0.5–1.0)
Mardorf	Germany	M	20	25	45	1	0.30	0.30	2.10	11.2 (2.3-inf)	68.9 (0.8–346)
Seebeckwiesen		Se	20	36		1	0.33	0.32	2.56	37.1 (8.5 - inf)	8.5 (0.2–31.5)
Sjöhusen		S	20	31		0	0.30	0.29	2.16	28.1 (4 - inf)	inf (inf - inf)
Tvödora	Skåne	T	19	32	31	0	0.34	0.31	2.12	inf (10.1 - inf)	inf (inf - inf)
Räften		R	20	29		0	0.29	0.27	2.17	8 (2.1–51.9)	inf (inf - inf)
Österbybruk		AÖ	20	53		16	0.51	0.43	3.95	1.0 (0.8–1.2)	1.0 (0.7–1.3)
Valsbrunna	Uppsala	V	20	33	46	0	0.32	0.32	2.13	inf (203.4 - inf)	inf (inf - inf)
Holmsjön		H	20	20		1	0.14	0.13	1.62	inf (19.2 - inf)	inf (inf - inf)
Nydalasjön	Umeå	Ny	20	20	17	0	0.15	0.12	1.51	39 (19.6 -inf)	36.7 (0.0–184)
Besbyn		B	20	20		0	0.16	0.09	1.77	37 (2.7-inf)	4 (0.0–20)
Ernäs	Luleå	E	20	17	20	1	0.12	0.08	1.45	inf (0.6-inf)	3.4 (0.0–16.8)
Total			239			25	0.28	0.25	2.22		

The populations are ordered from South to North. n = number of individuals; NA = alleles within a population; As = alleles within a sampling area; PA = private alleles; H_o_ = observed heterozygosity, H_e_ = expected heterozygosity; AR = allelic richness; Ne = effective population size by linkagedisequilibrium method (Ne (LDNe)) and by coancestry method (CNe)

The microsatellite locus RCO8640 was found to be under diversifying selection and had very low diversity in the northernmost populations (Luleå). This locus is located inside the up-regulated transcription factor gene C/EBP involved in the thyroid hormone pathway [62, 63], which is linked with adaptive divergence in larval developmental rate [56, 57]. We found that RCO8640 was under selection in the southern cluster, possibly suggesting selection on development rate along the southern part of the gradient, as found previously at local and broader geographical scales in northern European anurans (e.g., [57, 80]). While we did not find significant diversifying selection in the northern cluster, F_ST_ was still high along the northern part of the gradient. It would be very interesting to link variation in this locus to phenotypic variation in development rate along the latitudinal gradient.

When analyses were made on all the populations we found evidence of diversifying selection on two (MHC II exon 2 and RCO8640) and signs of stabilizing selection on five loci. We find two reasons which might explain why we find a high number of loci under stabilizing selection: 1) microsatellites were developed using known sequences of coding genes (See; [56, 57], 2) the long gradient with a high global F_ST_ allows for a better the detection of stabilizing selection. When the populations were divided into a northern and southern cluster we found evidence for selection in the southern cluster (three cases of stabilizing and two cases of diversifying selection) while in the northern cluster we could only find signs of stabilizing selection on one locus. These patterns could reflect actual differences among regions in the relative importance of drift and selection but we advise caution when interpreting the results of this study. While drift is predicted to be more important in small and fragmented populations and selection is more important in large connected ones [3], we cannot entirely rule out the possibility that the detected patterns may be partly due to lower sample sizes and fewer population contrasts in the north. So while in line with predictions from population genetic theory, these results should be deemed as tentative.

### Combined effects of recolonization history, selection and drift

We suggest that genetic variation among the regions and populations can be explained by complex patterns of selection, drift and the two recolonization routes of Scandinavia since the last glaciation (see [81] for a similar example). Our results provide an example of a situation where the level of adaptive MHC II exon 2 diversity seems to be correlated with neutral diversity among populations. This is not always the case as depletion in overall genetic variation may or may not be correlated with the amount of adaptive genetic variation [4]. Here we found that overall F_ST_ and genetic diversity indexes (AR and H_E_) were substantially higher for the MHC locus compared to neutral microsatellite markers (even though correlated). These results suggest that the MHC II exon 2 locus is under diversifying selection and are in agreement with previous studies finding more differentiation in MHC than in neutral loci suggesting adaptation to local parasite faunas (e.g. [27, 78]. However, earlier studies on differentiation at MHC loci show substantial heterogeneity. Other studies have found no difference among population differentiation at MHC and neutral markers indicating the dominant role of drift, and yet others find no differentiation at the MHC indicating balancing selection maintaining genetic variation (see [82] for a recent example, summarized by [83]). There is a need for further studies of the processes shaping within and among population genetic variation in natural populations to further improve our understanding on how genetic variation is geographically portioned and distributed.

## Methods

### Sample collection and DNA extraction

*R. arvalis* has a broad Euroasiatic distribution, from the North Sea coast to Siberia [67]. It lives in diverse habitats, from forests to pastures, and breeds in semi-permanent to permanent ponds and lakes. The centre of the distribution of *Rana arvalis* is located in the area of eastern Germany and western Poland (see distribution map in Sillero et al. [84]). We collected *R. arvalis* eggs in five regions from northern Germany (Hanover) to northern Sweden (Luleå) in 2014 and 2015 (Table 1, Additional file 10: Table S6; Figure 2). The eggs were collected at two sites in each region, with the exception of northern Germany and southern Sweden (Skåne), where samples were collected from three sites. The average distance between collection sites in the same region was 20 km (range 8 to 50 km). The collection sites were ponds situated in flat terrain dominated by mixed forest, pastures and agricultural land. At each site we collected ca. ten eggs from each of 20 freshly laid clutches. The eggs were transported to Uppsala and kept in a climate room at 16 °C. After hatching the tadpoles (stage 25, [85]) were euthanized with an overdose of MS222, preserved in 96% ethanol and stored at 4 °C until DNA extraction.

Genomic DNA was extracted from 240 tadpoles (one individual/clutch) using a high salt extraction precipitation protocol (modified from [86]). Purity and concentration of DNA were determined with a NanoDrop® 2000, spectrophotometer and Qubit®3.0 fluorometer Quantitation Kit (Invitrogen™). Species verification was carried out by mtDNA cytochrome b amplification followed by the addition of *HaeIII* restriction enzyme [87]. Digestions by *HaeIII* produces different, easily distinguishable banding patterns in *R. arvalis* and *R. temporaria.*

### Microsatellite genotyping

We successfully genotyped all individuals at 18 microsatellite loci isolated from different *Rana* species, and tested another set of ten microsatellites without success (Additional file 1: Table S1). Some of the successfully genotyped loci are situated in or near coding regions, increasing the probability of these markers being under selection. PCR amplification was performed individually for each microsatellite. Reactions were performed in a final volume of 20 μl using an ABI 2720 thermal cycler. The PCRs were done using either the Type-it® Microsatellite PCR Kit (Qiagen®, Sollentuna, Sweden) or DreamTaq (Thermo Scientific) following the manufacturer’s instructions. Additional file 11: Table S7 specifies the used PCR type, the exact reaction mix and the thermocycling conditions for each PCR reaction. Prior to genotyping, the PCR products were diluted in water (1:10, 1:30 or 1:50). 1 μl was mixed with 9.8 μl Hi-DiTM Formamide (P/N 4311320, Applied Biosystems) and 0.2 μl size standard (GeneScanTM, 600 LIZ®, Thermo Scientific) and run on 3730xl DNA Analyzer (Applied Biosystems). Samples were genotyped using GeneMapper® Software 5 (Life TechnologiesTM).

### MHC II exon 2 gene

#### Single locus amplification and preparation for sequencing

We amplified the complete second exon (272 bp) of the single MHC II gene (corresponding to the β −2 domain) in *R. arvalis*. The primers ELF_1 (3′- GAGGTGATCCCTCCAGTCAGT-5′) and ELR_2 (3′-GCATAGCAGACGGAGGAGTC-5′) were designed based on primers sequences developed for *R. pretiosa* and *R. luteiventris* [88], amplifying a 338 bp fragment with the primers positioned in the flanking intron-exon region (Additional file 12: Figure S5). Both forward and reverse primers were modified for Illumina MiSeq sequencing with an individual 8 bp barcode and a sequence of three N (to facilitate cluster identification). Each amplicon was marked with an individual combination of a forward and a reverse barcode for identification. PCR reactions were conducted in a total volume of 20 μl containing 1 μl of genomic DNA, 2 μl of 10X Dream taq buffer (Thermo scientific lab), 0.4 μl of 2 mM of each dNTP, 0.5 μl of each 10 μM primer (ELF_1 and ELR_2, respectively), 1.5 μl of Bovine serum albumine (BSA; 5 mg/ml) and 0.25 μl of Dream taq DNA polymerase (5 U/μl, Thermo scientific lab) in deionized water. Thermocycling was performed on the ABI 2720 (Applied Biosystems®). An initial denaturation step of 3 min at 95 °C was followed by a touch-down procedure, consisting of 30 s at 95 °C, annealing for 30 s at temperatures decreasing from 63 to 58 °C during the first 5 cycles (one detrimental degree per cycle) and ending with an extension step at 72 °C for 1 min. Thereafter, 30 similar cycles with a consistent annealing temperature of 58 °C followed, and PCR products were stored at 4 °C. All amplifications were carried out using filter tips in separate (pre- and post-PCR) rooms, and negative controls were included in all amplifications to avoid contaminations. PCR products were run and visualized on a 1.5% agarose gel using gelgreen (BIOTIUM). To reduce the number of samples for subsequent purification, 3–9 PCR products with similar concentrations were pooled based on estimations from the gel image. These sample pools were run on 1.5% agarose gel, the target band was excised from the gel and extracted using the MinElute Gel Extraction Kit (Qiagen® Sollentuna, Sweden). The concentration of each sample pool was measured with Quant-iT PicoGreen dsDNA assay kit (Invitrogen Life Technologies, Stockholm, Sweden) on a fluorescence microplate reader (Ultra 384; Tecan Group Ltd., Männedorf, Switzerland). The final amplicon pooling was done according to the measured concentrations and consisted of equimolecular amounts of each sample. A total of eight final amplicon pools were generated, and libraries were prepared using the Illumina Truseq DNA PCR-Free Sample preparation kit (Illumina Inc., San Diego, CA). Four pools each were combined into a Miseq run, and sequencing of two Miseq 250 runs was carried out at the National Genomic Infrastructure (NGI), the SNP&SEQ Technology Platform hosted at SciLifeLab in Uppsala (Sweden).

#### Miseq data analyses

Sequencing data were extracted from the raw data and combined into single forward reads using FLASH (Magoč and Salzberg 2011), each of the eight amplicon pools were analyzed independently. In total, eight fastq files were generated and transformed into fasta (multifasta) files using Avalanche NextGen package (DNA Baser Sequence Assembler v4 (2013), Heracle BioSoft, www.DnaBaser.com). The jMHC software [89] was used to remove primer sequences and unique tags, and to generate alignments of all variants per amplicon. Generally, in MHC studies using NGS techniques rigorous quality control and filtering procedures have to be applied to distinguish PCR and sequencing artefacts from true MHC allele. In this study, however, the distinction of true alleles and artefacts was greatly simplified, for three reasons: 1) amplifying a single gene will only yield one or two true MHC alleles per amplicon, and the project was designed in a way that both 2) replication rate and 3) per amplicon coverage was markedly high.

We assigned the two most frequent variants within each amplicon as valid MHC alleles that occurred in at least 3% of the reads [52, 90]. In a single locus system, it is not expected that chimera are generated in higher frequencies than the two parental alleles, and hence, no chimeric sequence that could have been taken for a valid MHC alleles could be detected when thoroughly checking the sequences per amplicon by eye.

If only one sequence exceeded the 3% rule, the amplicon was scored as a homozygote. We discarded amplicons with <300 reads from the analysis for quality reasons. Among amplicons, valid MHC alleles had to be present in at least two amplicons (2-independent-PCR-criterion, [91]), therefore we replicated all amplicons that revealed unique MHC alleles in the second Miseq run, and ran every individual twice in the second Miseq run from independent PCRs. In addition, we used the DOC method, not assuming any specific number of loci to identify and estimate the number of alleles (*Ai*) per individual. This procedure is based on the break point in sequencing coverage between variants within each individual and avoids choosing a subjective threshold to separate true alleles from artefacts. In this procedure, variants are sorted top-down by coverage, followed by the calculation of the coverage break point (DOC statistic) around each variant. The variant with the highest DOC value is assumed to be the last true allele (see [65]).

All valid allele sequences were imported and aligned in MEGA 6.0 [92]. Allele sequences were extensively compared to other sequences for the same locus: giant spiny frog (*Quasipaa spinosa*; GenBank: KM390904.1) natterjack toad (*Epidalea calamita*; GenBank: HQ388291.1), mouse (*Mus musculus*; GenBank: JN948541.1) and turkey (*Meleagris gallopavo*; GenBank: DQ993255.2). According to the GT/AG rule [93] and in concordance with exon 2 in *Odorrana tormota* (JQ918829), the exon boundary was defined 16 bps downstream from intron-exon boundary determined in mouse. We removed the intron sequences for further analyses and ended up with a MHC II exon 2 fragment of 272 bps. Valid MHC exon 2 alleles were named following the nomenclature suggested by Klein [29]: a four digit abbreviation of the species name followed by gene*numeration, e.g. Raar_DAB*01.

### Data analyses

#### Outlier analyses

We used the programs Lositan [94] and BayeScan v. 2.01 [95] to identify loci under divergent or uniform selection. The null distribution of *F*_ST_ was simulated with 100,000 iterations implementing a stepwise mutation model (SMM) in Lositan. In Bayescan, outliers were detected by implementing the multinomial Dirichlet model. Outliers identified by at least one of the two methods were deemed as being non-neutral (Additional file 2: Table S2), and the term “neutral loci” is used to refer to the non-outlier loci included in subsequent analyses.

The microsatellite and MHC II exon 2 data set were divided in different subdatasets: the entire gradient with all the populations included in the analyses; a northern cluster (Umeå and Luleå); and a southern cluster (Germany, Skåne and Uppsala). We did the division after analyzing our data (see below) and in accordance with a bidirectional recolonization hypothesis of Scandinavia [67, 68]. For MHC II exon 2 we transformed the sequence data into genotype data using PGBSpider [96] to be able to run the outlier test in the Lositan software while implementing a Stepwise Mutation Model (SMM) (see Fig. 1; Additional file 1: Table S1). We also ran the outlier analyses using an Infinite Alleles Model (IAM). The results identifying outlier loci were independent of the mutation rate model, IAM or SMM (see Additional file 13: Figure S6). Outlier analyses were also repeated excluding the German populations from the data set (See Additional file 14: Table S8 and Additional file 15: Figure S7).

#### Analyses of microsatellite data

Input files were converted for different analyses program formats using the Excel add-in Microsatellite toolkit [97]. The frequency of null alleles was estimated with two different softwares: FreeNA [98] and Genepop [99]. To assess neutral genetic diversity, expected heterozygosity (H_E_), observed heterozygosity (H_O_) and allelic richness (AR), allele numbers rarified to the smallest sample size were calculated for each locality in FSTAT 2.9.3.2 [100] Multi-locus means were obtained using Microsatellite toolkit. Effective population size was estimated based on two different methods: the linkage disequilibrium method and the coancestry method using the software Ne estimator [101].

To examine population structure and differentiation, global and pair-wise F_ST_ between all the populations [102] were calculated according to Nei et al. [103], as implemented in the R-package hierfstat [104] We used the ENA correction described in [98] and F’_ST_/G’_ST_ corrections [105] for all the F_ST_ values when appropriate. We tested for deviation from Hardy-Weinberg equilibrium (HWE) in ARLEQUIN v. 3.5. [106] and tested for Isolation by distance (IBD) by calculating pair-wise F_ST_ and log distance (km) for every population pair. Pair-wise F_ST_ was transformed based on Slatkin’s (1995) data adjustment (F_ST_/(1-F_ST_)). The Euclidean distance matrix was estimated using the R package Geosphere (see Hijmans et al. [107]). To test if pair-wise F_ST_ was spatially auto-correlated, Mantel tests were performed in R running the package Adegenet [108].

We visualized the spatial structure of microsatellite data using discriminant analysis of principal components (DAPC) implemented in Adegenet [108]. The optimal number of clusters for the DAPC was chosen based on the lowest Bayesian Information Criterion (BIC) value for the different clustering solutions, which coincided with a sharp break in the curve of BIC values as a function of k. Genetic clustering was also analyzed using STRUCTURE [109] to find the most likely number of genetic clusters (K) and assign individuals to these clusters assuming an admixture model and correlated allele frequencies with 10,000 burn-in steps and 100,000 MCMC (Markov chain Monte Carlo) steps. We investigated the likelihood of various numbers of K (2–5), following the approach suggested by Evanno et al. [110]. To infer the most likely K we visualized and compared the different likelihood distribution plots and ΔK plots produced by STRUCTURE HARVESTER [111] Runs for all the K were averaged using the LargeGreedy algorithm in CLUMPP software [112], which aligns cluster assignment across replicate analyses and were then visualized with DISTRUCT [113]. We estimated and visualized the gene flow patterns between all pairs of population samples by using divMigrate-online [66], which can detect asymmetric gene flow patterns. We included 12 populations, used G_ST_ (as implemented in divMigrate-online) as the differentiation metric and set the filter threshold to 0.20; 0.30; 0.40, respectively. Confidence limits on gene flow estimates were determined by 1000 bootstrap replicates.

#### Analyses of MHC II exon 2

To assess genetic diversity in the MHC II exon 2 standard diversity indices (H_E_, H_O_, and allelic frequencies) were calculated for each locality in Arlequin v 3.5 [106] Allelic richness was calculated in FSTAT 2.9.3.2 [100]. Allele frequency plots were created in R using the “ggplot2” package [114].

Global population differentiation, HWE expected values and a pair-wise distance matrix for all the populations were computed in ARLEQUIN v. 3.5; we ran 1000 permutations to allow calculation of confidence limits. We tested for Isolation by distance (IBD; [115]), as described above for the microsatellite data.

#### Comparing differentially selected and neutral loci

To compare population differentiation among the differentially selected loci, as revealed by the outlier analyses (above), we plotted the standardized F’_ST_ for MHC exon 2 and RCO8640, respectively, against the differentiation for neutral microsatellites, G’_ST_ [105]. We then computed the restricted major axis regression slopes for the northern and southern population comparisons separately for each comparison with a differentially selected marker. We hypothesized that if population differentiation was stronger among southern populations, the slope of this regression would be steeper than among northern populations.

## Conclusion

In summary, populations were shown to be subjected to different selective regimes and combined with different historical demographic patterns affecting the strength of genetic drift, a complex pattern of differentiation have evolved along the gradient. Some loci are more divergent than expected by drift among populations due to diversifying selection while others are more uniform among populations due to stabilizing selection. Our data show a high number of MHC exon 2 alleles in comparison to other European amphibian species. Both overall and MHC genetic variation are lower at northern latitudes which suggest a high risk of extinction when confronted with emerging pathogens and climate change. These results emphasize the importance of latitudinal gradient studies in order to elucidate and understand the evolutionary processes shaping genetic variation among natural populations.

## Additional files

Additional file 1: Table S1. Microsatellites used in the study. (PDF 40 kb)

Additional file 2: Table S2. 16 microsatellites outlier analyses results from Lositan and Bayescan. (PDF 31 kb)

Additional file 3: Table S3. 15 microsatellites and MHC II exon 2 outlier analyses results from Lositan and Bayescan. Analyses were performed independently considering: all the gradient, northern populations as a cluster and southern populations as another cluster. (PDF 31 kb)

Additional file 4: Figure S1. Isolation-by-distance a) for MHC class II and b) for microsatellite (MS) shown as Slatkin’s linearized pair-wise F_ST_ “(F_ST_/(1-F_ST_))” as a function of the natural logarithm of distance (km) between locality pairs. MHC class II is represented by blue circles and MS are represented by black stars. (PDF 2099 kb)

Additional file 5: Figure S2. Results of STRUCTURE analyses using the Admixture model. Average individual assignment probability (y-axis) of individuals for four values of K. Sampled populations are given below the plot and country, region or province of origin is given above. Delta K and Mean estimation Ln probability of the data are shown for different values of K. (PDF 326 kb)

Additional file 6: Figure S3. Discriminant component analyses (DAPC) of 9 neutral microsatellites for all the individuals. All individuals from the north cluster together to the right of the figure. (PDF 1539 kb)

Additional file 7: Table S4. Pairwise FSTs. a) MHC gene FST pairwise comparisons and b) neutral microsatellite FST pairwise comparisons, respectively. (PDF 49 kb)

Additional file 8: Figure S4. Colour scheme for the allele distribution maps a) MHC gene and b) RCO8640 gene. (PDF 875 kb)

Additional file 9: Table S5. Relative allele frequency for all the MHC alleles found along the latitudinal gradient. N = number of individual per population, AS = Alleles within sampling areas. (PDF 101 kb)

Additional file 10: Table S6. Information about pond locations along the latitudinal gradient. (PDF 48 kb)

Additional file 11: Table S7. PCR conditions for a total volume of 10 μl reaction for the neutral microsatellites finally used in the study. (PDF 69 kb)

Additional file 12: Figure S5. a) Primers were modified for Illumina Miseq consisting of a 8 bps barcode assignment and a sequence of three N Barcodes represented in pink (forward direction; “5- > 3”) and in orange (reverse direction; 3- > 5″). The NNN sequence is shown in blue. Possible primer pair combinations are presented in two different boxes (brown and orange, respectively). Every forward primer was combined with 9 different reverse primers for every pool. A total of four pools were constructed in the study: blue (A), purple (B), orange (C) and red (D). b) Outline of the library preparation. (PDF 173 kb)

Additional file 13: Figure S6. Results of outlier analyses by Lositan software in MHC class II exon 2 and RCO8640 according to a) Infinite allele model approach (IAM) b) Stepwise mutation model approach (SMM). (PDF 140 kb)

Additional file 14: Table S8. 15 microsatellites and MHC II exon 2 outlier analyses results from Lositan and Bayescan. Analyses were performed independently considering: all the gradient and southern population, excluding all the German locations. Overall *F*ST for the gradient excluding all population from Germany was 0.35. (PDF 17 kb)

Additional file 15: Figure S7. Results of outlier analyses by Lositan software for 15 microsatellites neutral markers, MHC class II exon 2 and RCO8640 according to the Stepwise mutation model approach (SMM) and excluding all the German populations. (PDF 856 kb)
