# Supplementary material for: Drift, selection, or migration? Processes affecting genetic differentiation and variation along a latitudinal gradient in an amphibian
Source: BMC Evol Biol. 2017 Aug 14;17:189. doi: 10.1186/s12862-017-1022-z (PMC5557520; doi:10.1186/s12862-017-1022-z)
Supplement: Supplementary file 1 — Microsatellites used in the study. (PDF 40 kb) [file 12862_2017_1022_MOESM1_ESM.pdf]

Table S1. Microsatellites used in the study.

Microsatellites list	Primer sequence	GenBank accession nº	Literature source
**Neutral**			
R1atCa17	F:CCCAATGTTTTCGTTCAACC<br>R:TGATTAGGAACCCCTGTGTTG	AF327361	Garner *et al.* 2002
Rtempμ4	F:TCATGCAGGCAAAGGTCAGG<br>R:AGAACCGCTGGTGTCCCAAC	AF297975	Rowe *et al.* 2001
Rtempμ5	F:GATCCACGTATGAGGATAGTTCAG<br>R:AGCAGGACTTGTGGACTGCG	AF297976	Rowe *et al.* 2001
RCIDII	F: GCTGAATTTTGGAAGATGAG<br>R: CAGGAAGAGCAAAAGTTCC	L42815	Davey *et al.* 1995
EU334906 (EU_06)	F:AATAAACGTGCTCAGGAGTGTG<br>R: TGACCAGCTAAGATAAATCGCA	BFG004	Matsuba *et al.* 2009
EU334912 (EU_12)	F:CAGCTCTCAATAGGACAGGCTC<br>R:ATCAGAAGGGAAAGGGGATAAG	BFG014	Matsuba *et al.* 2009
EU334915 (EU_15)	F:GACCTCGATTAGCCAGAAGACA<br>R:GCCCAAGGAAACATTACACAAT	BFG021	Matsuba *et al.* 2009
EU334919 (EU_19)	F:GCTCCCATTCCTTCTCTCTTTC<br>R:TAAACACCTTGCGGAGTAAAGC	BFG028	Matsuba *et al.* 2009
EU334924 (EU_24)	F:TCTGGGACATTGAAGAAGGATT<br>R:AAATAGACTGGGCACGAAGGT	BFG038	Matsuba *et al.* 2009
**Stabilizing selection**			
RtCa25	F: GCCAGGGTATGTAAACTTATGAGC<br>R: GTTTCAAATGTATATTATTGGTGCAATG	AF447866	Garner *et al.* 2002
WRA1-160	F: TCAAGCCCTGCATTACGGTGTG<br>R: CTGAAACATTCCCTGCAGCAGCT	AJ419883	Arens 2002
RRD590	F: GATTAGTAACAATAAAGGCACATG<br>R: CTTTGGAGCAGGAGGTG	D78590	Nakamura 1996
R1atCa18	F:ACTGCTGCATCCAAATCTCC<br>R:GGGTATAGCCATACACCTGTGC	AF327356	Garner *et al.* 2002
RtμP	F: TCATCCCTGCTACCTAATC<br>R: AAGCAATCAGGAATAAGGGG	AF489582	Pidancier *et al* 2002
**Diversifying selection**			
RC08604	F: TTGGAGACCCTCAGGG<br>R: TCATGGAAGTGTCCTTATCAC	U08604	Chen *et al*. 1994

**Null alleles**			
EU334911(EU_11)	F:TCAGCAATAGCATCTCTAGGGC<br>R:TCTGCATGGGTTTACTCCAGAT	BFG010	Matsuba *et al.* 2009
RECALQ	F: GGAGGGTGAAGTCAACACT<br>R:ACATTTGAATATATAAACTAGTTAGATGC	X64324	Zorzato 1992
R1atCa41	F: TGGTCTGCAAACTCATTCAC<br>R: GCAACAATGGGACTAATATAAGTGAG	AF327362	Garner *et al.* 2002
**Tried but not used**			
WRA1-22	F: GCACTGTCCTGTGTAGGAAT<br>R: GTTGCTATGACACAGGGC	AJ419881	Arens 2002
WRA1-28	F: GCCAGACCGTGACATAAAT<br>R: TAACCCCTTCCATTGAGC	AJ419882	Arens 2002
WRA6-8	F: CTAATGGCATATTCCTGCTC<br>R: TCGTTTTTTGAAGTGGACTT	AJ419884	Arens 2002
Rtempµ9	AGCGCCATGCTTATGCTGAG<br>TTGATATTTGCTTGCGGGGC	AF297980	Rowe *et al*. 2001
RNTYR2	F: TGGGACTTTTGCTAGCTG<br>R: GCCATAGGCCATAGACG	D37779	Nishioka 1995
RtCa11	F: CCTCTCCAGTACCTTTTCAGG<br>R: GCCCCCAGTCAATAGATTATG	AF447862	Garner *et al*. 2002
Rtempµ7	F:TCTTTCCTGCAACCCGTGTG<br>R:CCTTGTCCCTCTGGCAAAGC	AF297978	Rowe *et al*. 2001
RtµJ	F: TGGTCAACAACACACGGAAAGC<br>R: GATGTATTTGTCTCTGCTGCC	AF489580	Pidancier *et al* 2002
RNTYR2	F: TGGGACTTTTGCTAGCTG<br>R: GCCATAGGCCATAGACG	D37779	Nishioka 1995
Rt2Ca9	F:TCCCTTAATTGTGCATTTTTCC<br>R:CTCTTTTCCCTTATAGGTGCAG	AF327358.	Garner *et al*. 2002
