# Supplementary material for: Drift, selection, or migration? Processes affecting genetic differentiation and variation along a latitudinal gradient in an amphibian
Source: BMC Evol Biol. 2017 Aug 14;17:189. doi: 10.1186/s12862-017-1022-z (PMC5557520; doi:10.1186/s12862-017-1022-z)
Supplement: Supplementary file 2 — 16 microsatellites outlier analyses results from Lositan and Bayescan. (PDF 31 kb) [file 12862_2017_1022_MOESM2_ESM.pdf]

Table S2: 16 microsatellites outlier analyses results from Lositan and Bayescan.

	All gradient	
	**Lositan**	**Bayescan**
**Loci**		
**EU_06a**	*neutral*	*neutral*
**EU_12a**	*neutral*	*neutral*
**Eu_15a**	*neutral*	*neutral*
**EU_19a**	*neutral*	*neutral*
**EU_24a**	*neutral*	*neutral*
**Rtemp4a**	*neutral*	*neutral*
**Rtemp5a**	*neutral*	*neutral*
**RtCa17a**	*neutral*	*neutral*
**RD101a**	*neutral*	*neutral*
**RRDD590a**	*Stabilizing selection*	_
**RtuP**	*Stabilizing selection*	*Stabilizing selection*
**WRA_160**	*Stabilizing selection*	*Stabilizing selection*
**RCO8640**	*Diversifying selection*	*Diversifying selection*
**RtCa25**	_	*Diversifying selection*
**RtCAa18**	_	*Stabilizing selection*
