# Supplementary material for: Drift, selection, or migration? Processes affecting genetic differentiation and variation along a latitudinal gradient in an amphibian
Source: BMC Evol Biol. 2017 Aug 14;17:189. doi: 10.1186/s12862-017-1022-z (PMC5557520; doi:10.1186/s12862-017-1022-z)
Supplement: Supplementary file 3 — 15 microsatellites and MHC II exon 2 outlier analyses results from Lositan and Bayescan. Analyses were performed independently considering: all the gradient, northern populations as a cluster and southern populations as another cluster. (PDF 31 kb) [file 12862_2017_1022_MOESM3_ESM.pdf]

Table S3. 15 microsatellites and MHC II exon 2 outlier analyses results from Lositan and Bayescan. Analyses were performed independently considering: all the gradient, northern populations as a cluster and southern populations as another cluster.

	**Nothern cluster**		**Southern cluster**		**Gradient**	
	**Lositan**	**Bayescan**	**Lositan**	**Bayescan**	**Lositan**	**Bayescan**
**Loci**						
**RDD590**	–	_	–	_	*Stabilizing selection*	_
**RtuP**	*Stabilizing selection*	_	_	*Stabilizing selection*	*Stabilizing selection*	*Stabilizing selection*
**WRA_160**	–	–	–	_	*Stabilizing selection*	_
**RCO8640**	–	_	*Diversifying selection*	_	*Diversifying selection*	*Diversifying selection*
**MHC**	_	_	*Diversifying selection*	_	*Diversifying selection*	_
**RtCa25**	–	–	–	*Stabilizing selection*	_	*Stabilizing selection*
**RtCAa18**	–	–	–	*Stabilizing selection*	_	*Stabilizing selection*
