# Supplementary figures and images for: Drift, selection, or migration? Processes affecting genetic differentiation and variation along a latitudinal gradient in an amphibian
Source: BMC Evol Biol. 2017 Aug 14;17:189. doi: 10.1186/s12862-017-1022-z (PMC5557520; doi:10.1186/s12862-017-1022-z)

a)

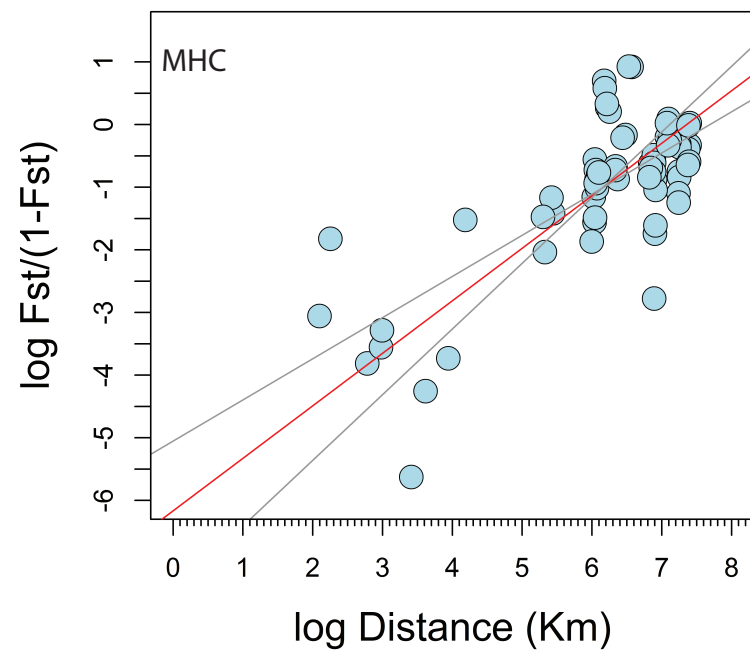

b)

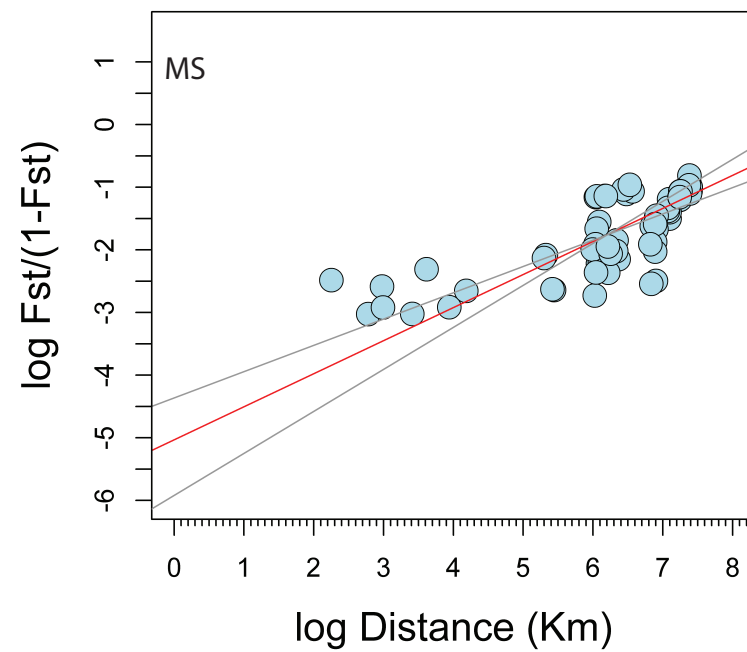

Supplement: Supplementary file 4 — Isolation-by-distance a) for MHC class II and b) for microsatellite (MS) shown as Slatkin’s linearized pair-wise FST “(FST/(1-FST))” as a function of the natural logarithm of distance (km) between locality pairs. MHC class II is represented by blue circles and MS are represented by black stars. (PDF 2099 kb) [file 12862_2017_1022_MOESM4_ESM.pdf]

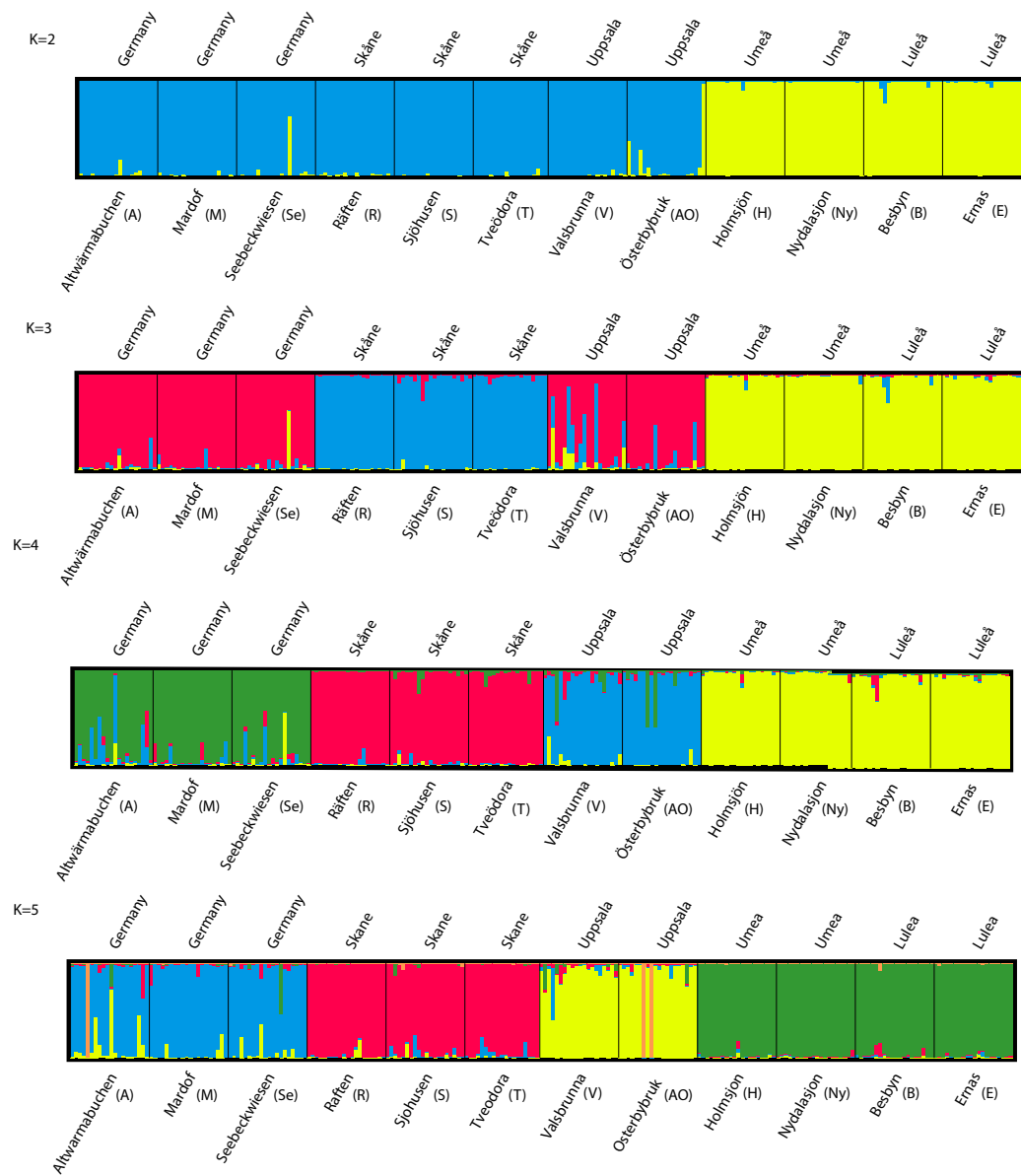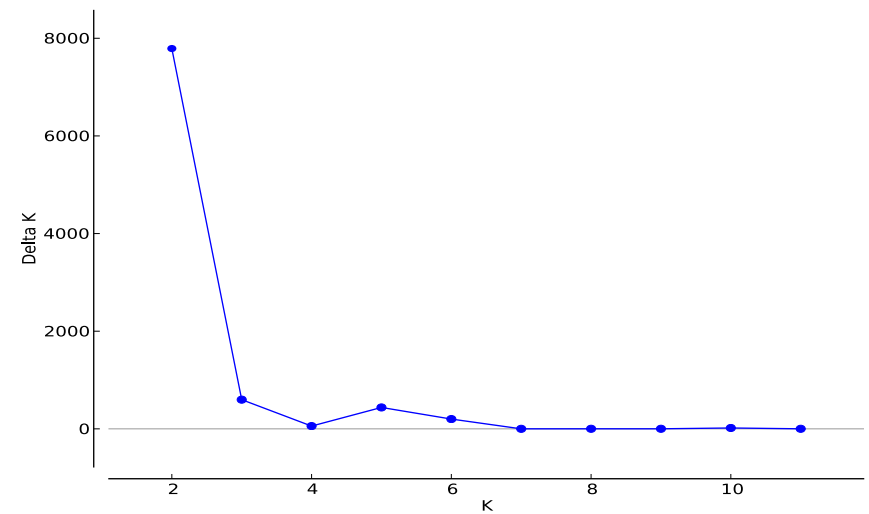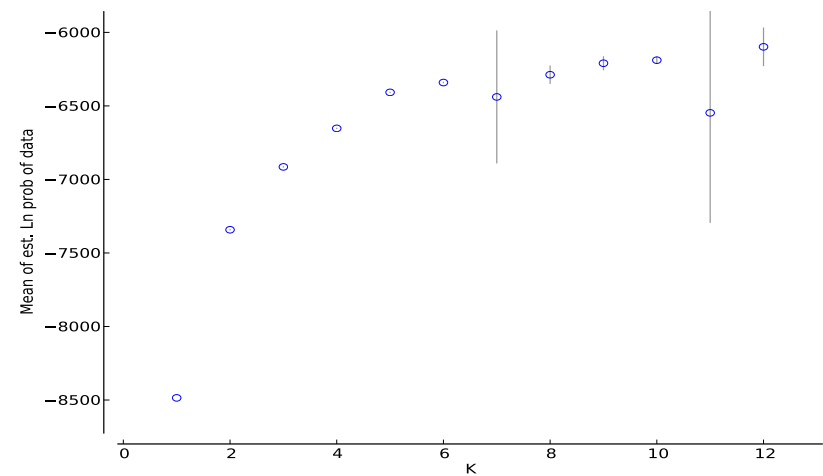

Supplement: Supplementary file 5 — Results of STRUCTURE analyses using the Admixture model. Average individual assignment probability (y-axis) of individuals for four values of K. Sampled populations are given below the plot and country, region or province of origin is given above. Delta K and Mean estimation Ln probability of the data are shown for different values of K. (PDF 326 kb) [file 12862_2017_1022_MOESM5_ESM.pdf]

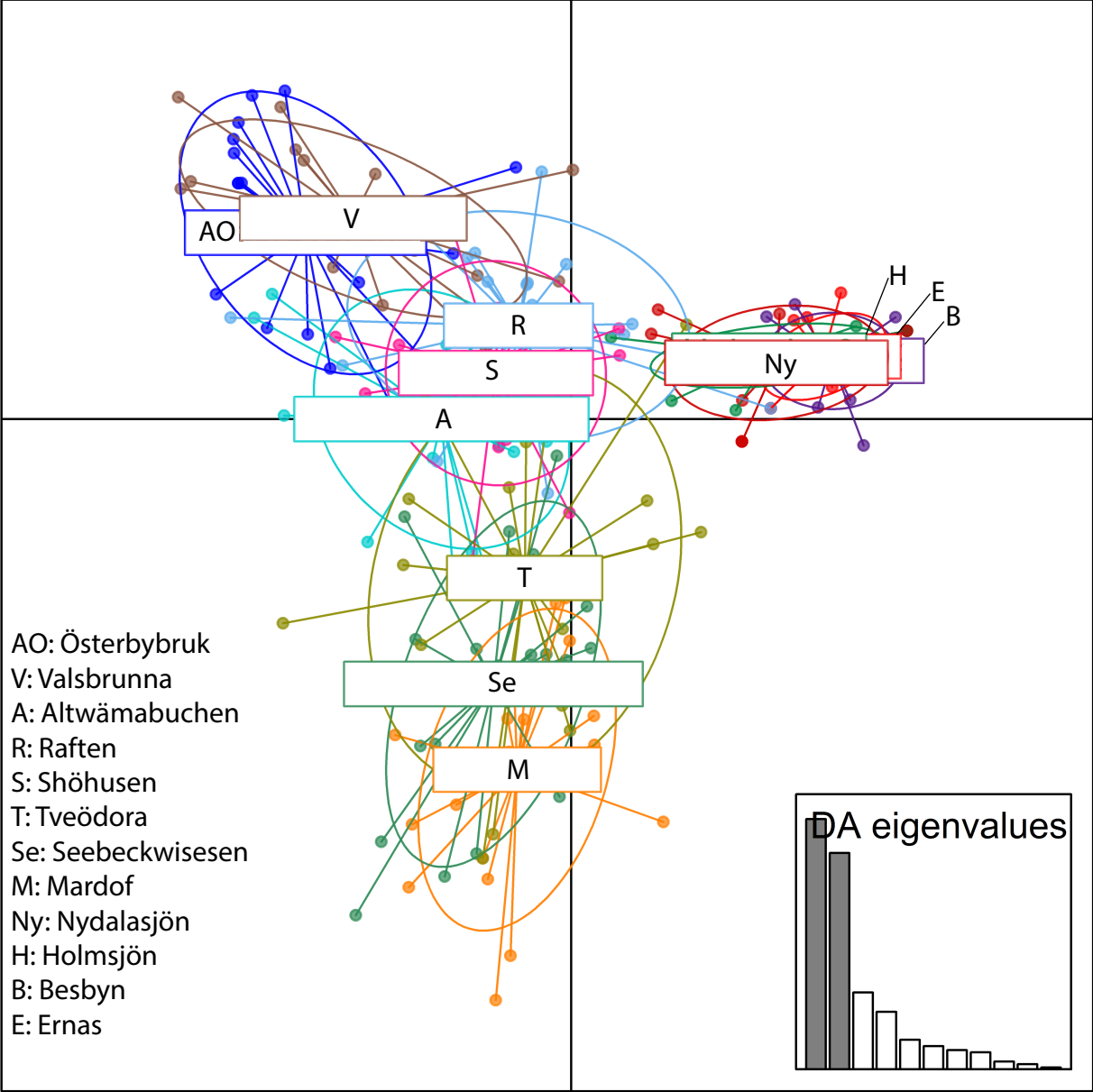

Supplement: Supplementary file 6 — Discriminant component analyses (DAPC) of 9 neutral microsatellites for all the individuals. All individuals from the north cluster together to the right of the figure. (PDF 1539 kb) [file 12862_2017_1022_MOESM6_ESM.pdf]

a) PCR step I

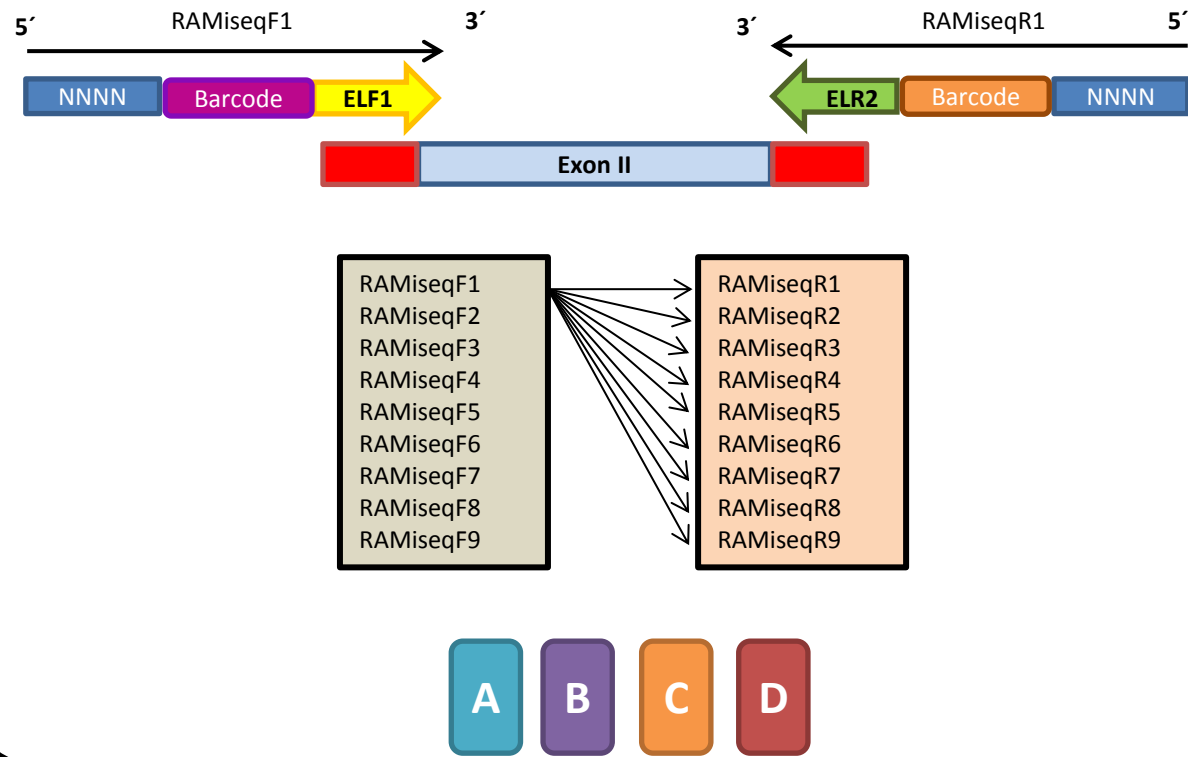

b) Library preparation Step II

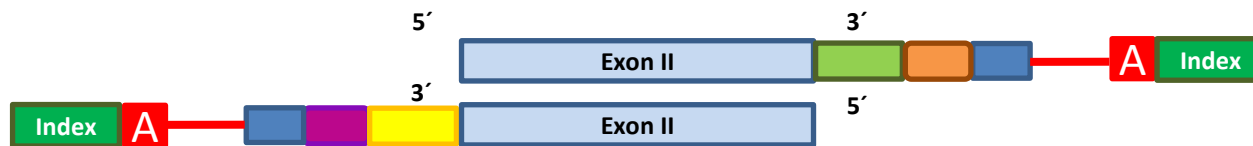

Supplement: Supplementary file 12 — a) Primers were modified for Illumina Miseq consisting of a 8 bps barcode assignment and a sequence of three N Barcodes represented in pink (forward direction; “5- > 3”) and in orange (reverse direction; 3- > 5″). The NNN sequence is shown in blue. Possible primer pair combinations are presented in two different boxes (brown and orange, respectively). Every forward primer was combined with 9 different reverse primers for every pool. A total of four pools were constructed in the study: blue (A), purple (B), orange (C) and red (D). b) Outline of the library preparation. (PDF 173 kb) [file 12862_2017_1022_MOESM12_ESM.pdf]

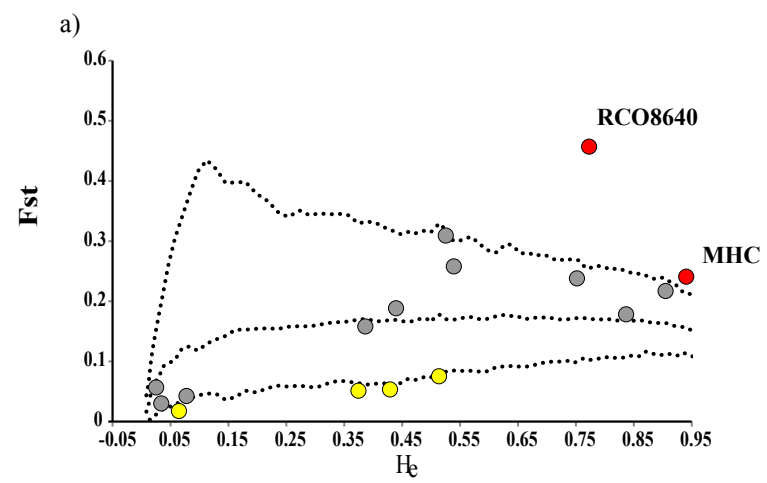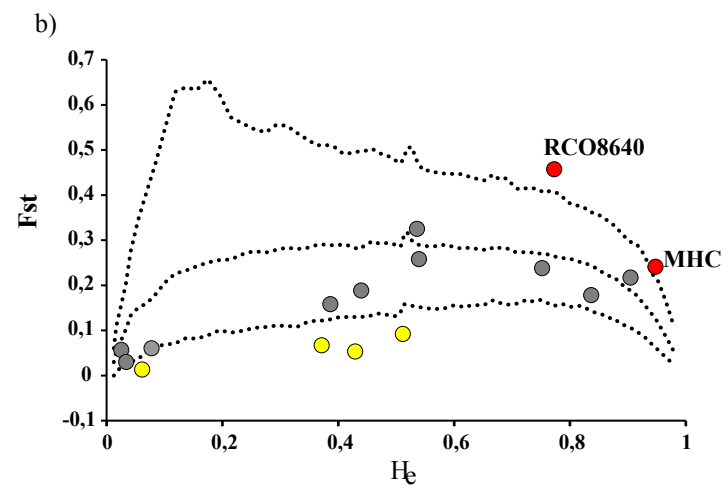

Supplement: Supplementary file 13 — Results of outlier analyses by Lositan software in MHC class II exon 2 and RCO8640 according to a) Infinite allele model approach (IAM) b) Stepwise mutation model approach (SMM). (PDF 140 kb) [file 12862_2017_1022_MOESM13_ESM.pdf]

a)

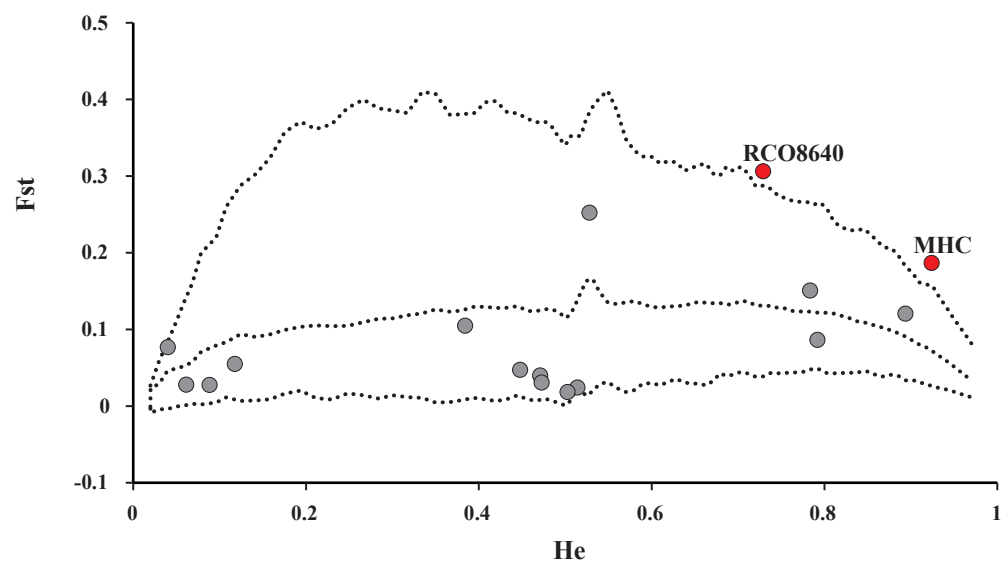

b)

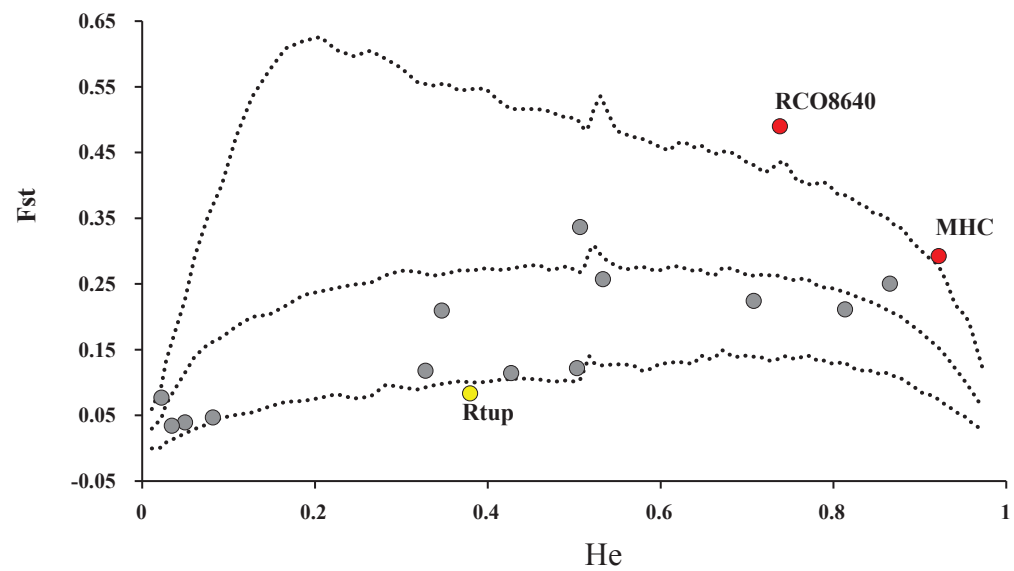

Supplement: Supplementary file 15 — Results of outlier analyses by Lositan software for 15 microsatellites neutral markers, MHC class II exon 2 and RCO8640 according to the Stepwise mutation model approach (SMM) and excluding all the German populations. (PDF 856 kb) [file 12862_2017_1022_MOESM15_ESM.pdf]
