# Supplementary material for: Drift, selection, or migration? Processes affecting genetic differentiation and variation along a latitudinal gradient in an amphibian
Source: BMC Evol Biol. 2017 Aug 14;17:189. doi: 10.1186/s12862-017-1022-z (PMC5557520; doi:10.1186/s12862-017-1022-z)
Supplement: Supplementary file 7 — Pairwise FSTs. a) MHC gene FST pairwise comparisons and b) neutral microsatellite FST pairwise comparisons, respectively. (PDF 49 kb) [file 12862_2017_1022_MOESM7_ESM.pdf]

Table S4. Pairwise F_{ST}s. a) MHC gene F_{ST} pairwise comparisons and b) neutral microsatellite F_{ST} pairwise comparisons, respectively.

a)	**A**	**B**	**E**	**H**	**M**	**Ny**	**AÖ**	**R**	**Se**	**S**	**T**	**V**
**A**	0.000	*										
**B**	0.418	0.000	*									
**E**	0.404	0.004	0.000	*								
**H**	0.322	0.195	0.116	0.000	*							
**M**	0.014	0.506	0.497	0.421	0.000	*						
**Ny**	0.302	0.237	0.186	0.045	0.411	0.000	*					
**AÖ**	0.149	0.459	0.448	0.364	0.167	0.326	0.000	*				
**R**	0.293	0.458	0.450	0.366	0.312	0.352	0.339	0.000	*			
**Se**	0.022	0.356	0.344	0.250	0.023	0.223	0.059	0.174	0.000	*		
**S**	0.240	0.438	0.418	0.294	0.282	0.261	0.295	0.028	0.134	0.000	*	
**T**	0.266	0.523	0.506	0.380	0.317	0.320	0.326	0.139	0.184	0.036	0.000	*
**V**	0.349	0.715	0.716	0.668	0.333	0.641	0.179	0.567	0.301	0.552	0.582	0.000

b)	A	B	E	H	M	Ny	AÖ	R	Se	S	T	V
**A**	0.000	*										
**B**	0.311	0.000	*									
**E**	0.295	0.053	0.000	*								
**H**	0.202	0.145	0.129	0.000	*							
**M**	0.048	0.321	0.311	0.208	0.000	*						
**Ny**	0.227	0.150	0.171	0.031	0.246	0.000	*					
**AÖ**	0.072	0.299	0.268	0.189	0.109	0.219	0.000	*				
**R**	0.129	0.332	0.283	0.227	0.150	0.256	0.136	0.000	*			
**Se**	0.031	0.293	0.283	0.213	0.036	0.238	0.093	0.136	0.000	*		
**S**	0.116	0.338	0.286	0.231	0.133	0.257	0.115	0.043	0.123	0.000	*	
**T**	0.122	0.325	0.294	0.230	0.107	0.251	0.122	0.040	0.102	0.031	0.000	*
**V**	0.073	0.306	0.268	0.163	0.104	0.184	0.037	0.098	0.093	0.101	0.109	0.000
