# Supplementary material for: Drift, selection, or migration? Processes affecting genetic differentiation and variation along a latitudinal gradient in an amphibian
Source: BMC Evol Biol. 2017 Aug 14;17:189. doi: 10.1186/s12862-017-1022-z (PMC5557520; doi:10.1186/s12862-017-1022-z)
Supplement: Supplementary file 8 — Colour scheme for the allele distribution maps a) MHC gene and b) RCO8640 gene. (PDF 875 kb) [file 12862_2017_1022_MOESM8_ESM.pdf]

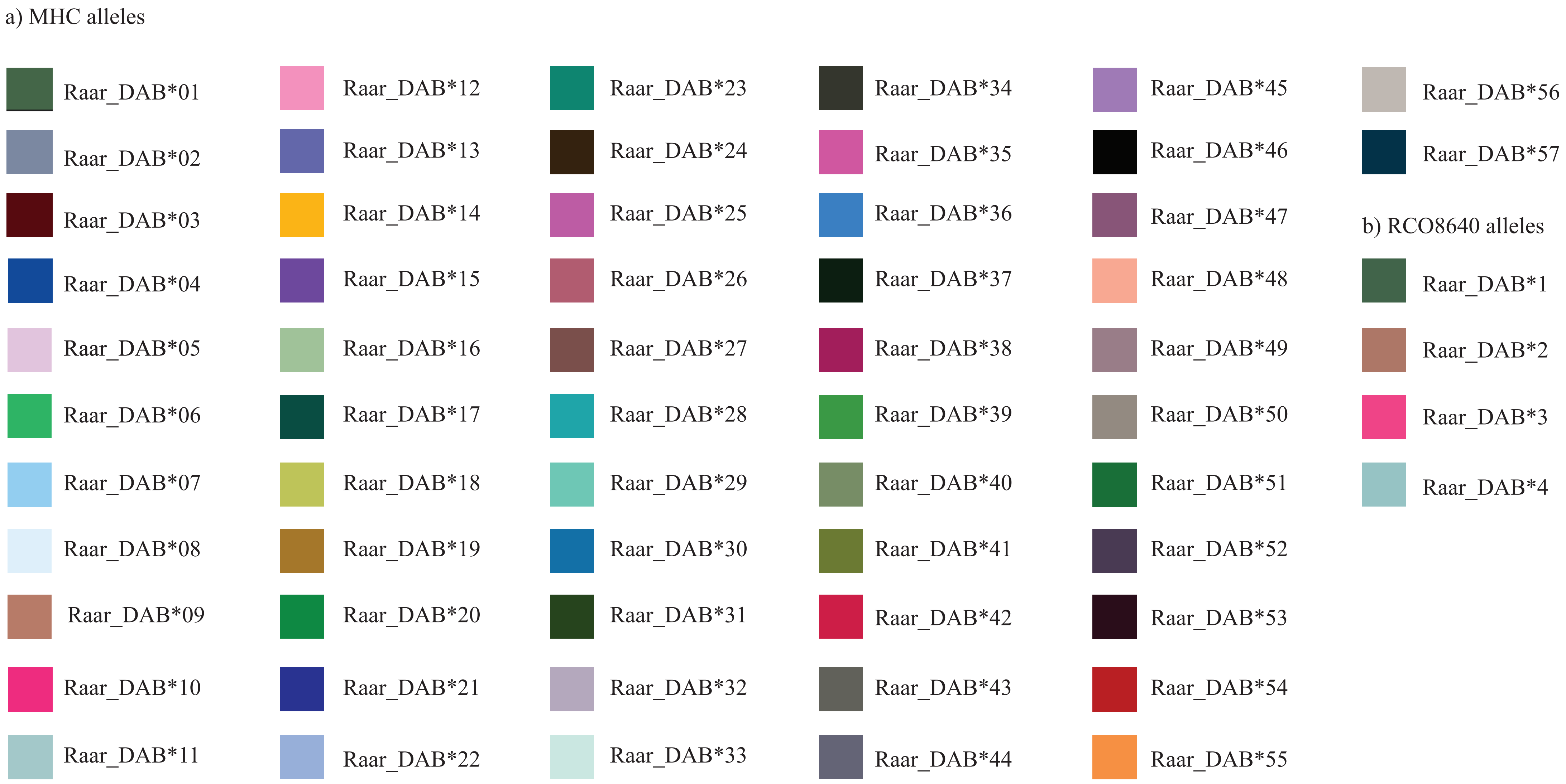

a) MHC alleles
Raar_DAB*01
Raar_DAB*02
Raar_DAB*03
Raar_DAB*04
Raar_DAB*05
Raar_DAB*06
Raar_DAB*07
Raar_DAB*08
Raar_DAB*09
Raar_DAB*10
Raar_DAB*11
Raar_DAB*12
Raar_DAB*13
Raar_DAB*14
Raar_DAB*15
Raar_DAB*16
Raar_DAB*17
Raar_DAB*18
Raar_DAB*19
Raar_DAB*20
Raar_DAB*21
Raar_DAB*22
Raar_DAB*23
Raar_DAB*24
Raar_DAB*25
Raar_DAB*26
Raar_DAB*27
Raar_DAB*28
Raar_DAB*29
Raar_DAB*30
Raar_DAB*31
Raar_DAB*32
Raar_DAB*33
Raar_DAB*34
Raar_DAB*35
Raar_DAB*36
Raar_DAB*37
Raar_DAB*38
Raar_DAB*39
Raar_DAB*40
Raar_DAB*41
Raar_DAB*42
Raar_DAB*43
Raar_DAB*44
Raar_DAB*45
Raar_DAB*46
Raar_DAB*47
Raar_DAB*48
Raar_DAB*49
Raar_DAB*50
Raar_DAB*51
Raar_DAB*52
Raar_DAB*53
Raar_DAB*54
Raar_DAB*55
Raar_DAB*56
Raar_DAB*57
b) RCO8640 alleles
Raar_DAB*1
Raar_DAB*2
Raar_DAB*3
Raar_DAB*4
