# Supplementary material for: Drift, selection, or migration? Processes affecting genetic differentiation and variation along a latitudinal gradient in an amphibian
Source: BMC Evol Biol. 2017 Aug 14;17:189. doi: 10.1186/s12862-017-1022-z (PMC5557520; doi:10.1186/s12862-017-1022-z)
Supplement: Supplementary file 9 — Relative allele frequency for all the MHC alleles found along the latitudinal gradient. N = number of individual per population, AS = Alleles within sampling areas. (PDF 101 kb) [file 12862_2017_1022_MOESM9_ESM.pdf]

Table S5. Relative allele frequency for all the MHC alleles found along the latitudinal gradient. N= number of individual per population, A_S = Alleles within sampling areas.

A_s	Germany			Skane			Uppsala		Umeå		Luleå	
**N**	14	10	10	20	20	19	18	19	19	19	20	19
**Raar_DAB*01**	**0.071**	0	0	0	0	0	0	0	0	0	0	0
**Raar_DAB*02**	**0.321**	**0.1**	**0.1**	0	0	0	0	0	0	0	0	0
**Raar_DAB*03**	**0.107**	0	0	0	0	0	0	0	0	0	0	0
**Raar_DAB*04**	**0.143**	**0.1**	**0.15**	0	0	0	0	0	0	0	0	0
**Raar_DAB*05**	**0.071**	**0.2**	**0.15**	0	0	0	0	0	0	0	0	0
**Raar_DAB*06**	**0.036**	0	0	0	**0.05**	0	0	0	0	0	0	0
**Raar_DAB*07**	**0.143**	0	0	0	0	0	0	0	0	0	0	0
**Raar_DAB*08**	**0.107**	0	**0.05**	0	0	0	0	0	0	0	0	0
**Raar_DAB*09**	0	0	0	0	0	0	**0.167**	0	**0.026**	**0.026**	0	0
**Raar_DAB*10**	0	0	0	0	0	0	**0.028**	0	0	0	0	0
**Raar_DAB*11**	0	0	0	0	0	0	**0.028**	0	0	0	0	0
**Raar_DAB*12**	0	0	0	0	0	0	**0.139**	**0.026**	0	0	0	0
**Raar_DAB*13**	0	0	0	0	0	0	**0.056**	**0.026**	0	0	0	0
**Raar_DAB*14**	0	**0.05**	0	0	0	0	**0.25**	0	0	0	0	0
**Raar_DAB*15**	0	**0.2**	**0.2**	**0.05**	**0.125**	**0.053**	**0.167**	0	**0.105**	**0.289**	0	0
**Raar_DAB*16**	0	0	0	0	0	0	**0.028**	0	0	0	0	0
**Raar_DAB*17**	0	0	0	0	0	0	**0.028**	**0.421**	0	0	0	0
**Raar_DAB*18**	0	0	0	0	0	0	**0.028**	0	0	**0.053**	0	0
**Raar_DAB*19**	0	0	0	0	0	0	**0.083**	0	0	0	0	0
**Raar_DAB*20**	0	0	0	0	0	0	0	0	**0.447**	**0.395**	**0.825**	**0.711**
**Raar_DAB*21**	0	0	0	0	0	0	0	0	**0.395**	**0.053**	**0.125**	**0.237**
**Raar_DAB*22**	0	0	0	0	0	**0.053**	0	0	**0.026**	0	0	0
**Raar_DAB*23**	0	0	0	0	0	0	0	0	0	**0.026**	0	0
**Raar_DAB*24**	0	0	0	0	0	0	0	0	0	**0.105**	0	0
**Raar_DAB*25**	0	0	0	0	0	0	0	0	0	**0.026**	0	0
**Raar_DAB*26**	0	0	0	0	**0.025**	0	0	0	0	**0.026**	0	0
**Raar_DAB*27**	0	0	0	**0.45**	**0.075**	**0.184**	0	0	0	0	0	0
**Raar_DAB*28**	0	0	**0.1**	**0.35**	**0.325**	**0.658**	0	0	0	0	0	0
**Raar_DAB*29**	0	0	0	**0.075**	0	0	0	0	0	0	0	0
**Raar_DAB*30**	0	0	0	**0.05**	0	0	0	0	0	0	0	0
**Raar_DAB*31**	0	0	0	**0.025**	**0.05**	**0**	0	0	0	0	0	0
**Raar_DAB*32**	0	0	0	0	0	0	0	0	0	0	**0.025**	0
**Raar_DAB*33**	0	0	0	0	0	0	0	0	0	0	**0.025**	0
**Raar_DAB*34**	0	0	0	0	**0.1**	0	0	0	0	0	0	0
**Raar_DAB*35**	0	0	0	0	**0.025**	0	0	0	0	0	0	0
**Raar_DAB*36**	0	0	0	0	**0.025**	0	0	0	0	0	0	0
**Raar_DAB*37**	0	0	0	0	**0.05**	**0.026**	0	0	0	0	0	0
**Raar_DAB*38**	0	0	0	0	**0.025**	0	0	0	0	0	0	0
**Raar_DAB*39**	0	0	0	0	**0.025**	0	0	0	0	0	0	0
**Raar_DAB*40**	0	0	0	0	**0.05**	0	0	0	0	0	0	0

**Raar_DAB*41**	0	0	0	0	**0.05**	0	0	0	0	0	0	0
**Raar_DAB*42**	0	0	0	0	0	0	0	0	0	0	0	**0.026**
**Raar_DAB*43**	0	0	0	0	0	0	0	0	0	0	0	**0.026**
**Raar_DAB*44**	0	0	**0.05**	0	0	0	0	**0.132**	0	0	0	0
**Raar_DAB*45**	0	0	0	0	0	0	0	**0.053**	0	0	0	0
**Raar_DAB*46**	0	0	0	0	0	0	0	**0.079**	0	0	0	0
**Raar_DAB*47**	0	0	0	0	0	0	0	**0.053**	0	0	0	0
**Raar_DAB*48**	0	0	0	0	0	0	0	**0.026**	0	0	0	0
**Raar_DAB*49**	0	0	0	0	0	0	0	**0.026**	0	0	0	0
**Raar_DAB*50**	0	0	0	0	0	0	0	**0.026**	0	0	0	0
**Raar_DAB*51**	0	0	0	0	0	0	0	**0.132**	0	0	0	0
**Raar_DAB*52**	0	0	0	0	0	**0.026**	0	0	0	0	0	0
**Raar_DAB*53**	0	**0.25**	0	0	0	0	0	0	0	0	0	0
**Raar_DAB*54**	0	**0.05**	0	0	0	0	0	0	0	0	0	0
**Raar_DAB*55**	0	**0.05**	0	0	0	0	0	0	0	0	0	0
**Raar_DAB*56**	0	0	**0.1**	0	0	0	0	0	0	0	0	0
**Raar_DAB*57**	0	0	**0.1**	0	0	0	0	0	0	0	0	0
