# Supplementary material for: Drift, selection, or migration? Processes affecting genetic differentiation and variation along a latitudinal gradient in an amphibian
Source: BMC Evol Biol. 2017 Aug 14;17:189. doi: 10.1186/s12862-017-1022-z (PMC5557520; doi:10.1186/s12862-017-1022-z)
Supplement: Supplementary file 10 — Information about pond locations along the latitudinal gradient. (PDF 48 kb) [file 12862_2017_1022_MOESM10_ESM.pdf]

Table S6. Information about pond locations along the latitudinal gradient.

Locality	Sampling area	code	Coordinates	
**Altwarmbüchen**		A	52°25'15.81"N	9°51'27.71"E
**Mardorf**	Germany	M	52°30'31.2"N	9°19'44.4"E
**Seebeekwiesen**		Se	52°27'52.9"N	10°05'03.8"E
**Sjöhusen**		R	55°32'42.40"N	13°16'24.53"E
**Tvedöra**	Skåne	S	55°42'0.81"N	13°25'51.54"E
**Räften**		T	55°43'17.34"N	13°17'3.02"E
**Österbybruk**	Uppsala	AÖ	60°10'42.09"N	17°51'16.06"E
**Valsbrunna**		V	59°45'14.66"N	17° 2'8.95"E
**Holmsjön**	Umeå	H	63°45'27.21"N	20°24'22.02"E
**Nydalasjön**		Ny	63°49'23.44"N	20°20'1.60"E
**Besbyn**		B	65°41'5.19"N	22°12'43.80"E
**Ernäs**	Luleå	E	65°31'14.69"N	21°41'12.78"E
