# Supplementary material for: Drift, selection, or migration? Processes affecting genetic differentiation and variation along a latitudinal gradient in an amphibian
Source: BMC Evol Biol. 2017 Aug 14;17:189. doi: 10.1186/s12862-017-1022-z (PMC5557520; doi:10.1186/s12862-017-1022-z)
Supplement: Supplementary file 11 — PCR conditions for a total volume of 10 μl reaction for the neutral microsatellites finally used in the study. (PDF 69 kb) [file 12862_2017_1022_MOESM11_ESM.pdf]

Table S7. PCR conditions for a total volume of 10 µl reaction for the neutral microsatellites finally used in the study

				PCR Mix volumes (µl)											
**Microsatellite Name**	**Repeat Unit**	**Size**	**PCR type**	**MgCl2 (50mM)**	**ddH20**	**Q solution (5x)**	**BSA (20mg/ml)**	**Buffer**	**Taq**	**Primer (10µM)**	**dNTPs (10mM)**	**Genomic DNA**	**Ta**	**cycles**	**Touch down**
**EU334906 (EU_06)**	(GT)9	133–157	Dream taq (5Units/ul)	_	7	_	0.75	1	0.05	0.25	0.2	1	57	35	_
**EU334912 (EU_12)**	(AC)21	93–141	Dream taq (5Units/ul)	_	7	_	0.75	1	0.05	0.25	0.2	1	57	35	_
**EU334915 (EU_15)**	(GT)11	137–161	Dream taq (5Units/ul)	_	7	_	0.75	1	0.05	0.25	0.2	1	58	35	_
**EU334919 (EU_19)**	(CT)8	277–283	Dream taq (5Units/ul)	_	7	_	0.75	1	0.05	0.25	0.2	1	59	35	_
**EU334924 (EU_24)**	(AG)16	176–196	type it (2x)	_	1	1	_	_	5	1	_	1	52	35	_
**Rtempµ4**	(AC)16	106–142	Dream taq (5Units/ul)	_	7	_	0.75	1	0.05	0.25	0.2	1	62	35	_
**RCIDII**	(TA)9	173	Dream taq (5Units/ul)	_	7	_	0.75	1	0.05	0.25	0.2	1	44	35	_
**R1atCa17**	(CA)8(C)10	113	type it (2x)	____	1	1	_	_	5	1	_	1	52	35	_
**Rtempµ5**	(GT)15	117–157	type it (2x)	_	1	1	_	_	5	1	_	2	53	35	_
**RECALQ**	(GAT/C)42	208	Fermentas (5Units/ul)	1,35	7	_	_	1	0.05	0,2	0,2	1	48	35	53-48
**RtCa25**	(TA)7(CA)5AA(CA)13T(AC)6	137	Fermentas (5Units/ul)	1,35	7	_	_	1	0.05	0,2	0,2	1	57	35	62-57
**RtuP**	(TC)6	173	Dream taq (5Units/ul)	_	7	_	0.75	1	0.05	0.25	0.2	1	44	35	_
**RtCa18**	(CA)12(N†)13(G)13	111	Fermentas (5Units/ul)	1,35	7	_	_	1	0.05	0,2	0,2	1	60	35	_
**RtCa41**	(CA)1(TA)1(CA)7(CG)1(CA)2	154	type it (2x)	_	1	1	_	_	5	1	_	1	52	35	_
**RDD590**	(GAA)3(GAG)13	176	type it (2x)	_	1	1	_	_	5	1	_	1	54	35	_
**EU334911(EU_11)**	(AC)19	150-350	Dream taq (5Units/ul)	_	7	_	0.75	1	0.05	0.25	0.2	1	57	35	_
**WRA_160**	(AC)11	494	Dream taq (5Units/ul)	_	7	_	0.75	1	0.05	0.25	0.2	1	54	35	_
**RCO8640**	(C)15	170-174	type it (2x)	_	1	1	_	_	5	1	_	2	55	35	_
