# Supplementary material for: Drift, selection, or migration? Processes affecting genetic differentiation and variation along a latitudinal gradient in an amphibian
Source: BMC Evol Biol. 2017 Aug 14;17:189. doi: 10.1186/s12862-017-1022-z (PMC5557520; doi:10.1186/s12862-017-1022-z)
Supplement: Supplementary file 14 — 15 microsatellites and MHC II exon 2 outlier analyses results from Lositan and Bayescan. Analyses were performed independently considering: all the gradient and southern population, excluding all the German locations. Overall FST for the gradient excluding all population from Germany was 0.35. (PDF 17 kb) [file 12862_2017_1022_MOESM14_ESM.pdf]

Table S8. 15 microsatellites and MHC II exon 2 outlier analyses results from Lositan and Bayescan. Analyses were performed independently considering: all the gradient and southern population, excluding all the German locations. Overall F_{ST} for the gradient excluding all population from Germany was 0.35.

	**Southern cluster**		**Gradient**	
**Loci**	**Lositan**	**Bayescan**	**Lositan**	**Bayescan**
**RtuP**	_	_	*Stabilizing selection*	*Stabilizing selection*
**WRA_160**	_	_	_	*Stabilizing selection*
**RCO8640**	*Diversifying selection*	_	*Diversifying selection*	*Diversifying selection*
**MHC**	*Diversifying selection*	_	*Diversifying selection*	_
**RtCa25**	_	*Stabilizing selection*	_	_
**RtCa18**	_	_	_	*Stabilizing selection*
